# Nutraceutical supplements in management of pain and disability in osteoarthritis: a systematic review and meta-analysis of randomized clinical trials

**DOI:** 10.1038/s41598-020-78075-x

**Published:** 2020-12-01

**Authors:** Dawood Aghamohammadi, Neda Dolatkhah, Fahimeh Bakhtiari, Fariba Eslamian, Maryam Hashemian

**Affiliations:** 1 Department of Anesthesiology, Faculty of Medicine, Tabriz University of Medical Sciences, Tabriz, Iran; 2 Physical Medicine and Rehabilitation Research Center, Aging Research Institute, Tabriz University of Medical Sciences, Emam Reza Hospital, Golgasht, Azadi Ave., Tabriz, Iran; 3 Department of Biology, School of Arts and Sciences, Utica College, Utica, USA

**Keywords:** Rheumatic diseases, Nutrition

## Abstract

This study designed to evaluate the effect of nutraceutical supplementation on pain intensity and physical function in patients with knee/hip OA. The MEDLINE, Web of Science, Cochrane Library, Scopus, EMBASE, Google Scholar, Science direct, and ProQuest in addition to SID, Magiran, and Iranmedex were searched up to March 2020. Records (n = 465) were screened via the PICOS criteria: participants were patients with hip or knee OA; intervention was different nutritional supplements; comparator was any comparator; the outcome was pain intensity (Visual analogue scale [VAS]) and physical function (Western Ontario and McMaster Universities Arthritis [WOMAC] index); study type was randomized controlled trials. The random effects model was used to pool the calculated effect sizes. The standardized mean difference (SMD) of the outcome changes was considered as the effect size. The random effects model was used to combine the effect sizes. Heterogeneity between studies was assessed by Cochran's (Q) and I2 statistics. A total of 42 RCTs were involved in the meta-analysis. Nutritional supplementation were found to improve total WOMAC index (SMD = − 0.23, 95% CI − 0.37 to − 0.08), WOMAC pain (SMD = − 0.36, 95% CI − 0.62 to − 0.10) and WOMAC stiffness (SMD = − 0.47, 95% CI − 0.71 to − 0.23) subscales and VAS (SMD = − 0.79, 95% CI − 1.05 to − 0.05). Results of subgroup analysis according to the supplementation duration showed that the pooled effect size in studies with < 10 months, 10–20 months and > 20 months supplementation duration were 0.05, 0.27, and 0.36, respectively for WOMAC total score, 0.14, 0.55 and 0.05, respectively for WOAMC pain subscale, 0.59, 0.47 and 0.41, respectively for WOMAC stiffness subscale, 0.05, 0.57 and 0.53, respectively for WOMAC physical function subscale and 0.65, 0.99 and 0.12, respectively for VAS pain. The result suggested that nutraceutical supplementation of patients with knee/hip OA may lead to an improvement in pain intensity and physical function.

## Introduction

Osteoarthritis (OA) as a degenerative chronic joint cartilage disorder is the most prevalent and principal reason for joint pain and functional impairment in the world^1^. OA is more prevalent in older adults and it will inflict incredible economic and societal charges and disturb life quality in different aspects subsequently in the future^2^. On the other hand, discomfort, pain and decreases in functional ability because of OA can consequence a greater risk of overweight/obesity, diabetes mellitus and falls and fractures^3^. Issues that chip into the development of OA consist of general factors (age, sex, overweight/obesity and nutrition) and local biomechanical factors (joint injury, physical activities and joint space)^4^.

Existing recommendations for the management of OA consist of three major classes: pharmacologic (i.e. opioids, non-steroidal anti-inflammatory drugs (NSAID), and COX-2 specific drugs), non-pharmacologic (i.e. rehabilitation to facilitate healthy body composition, lifestyle, and physical activity) and surgical treatment^4–7^. Present pharmacological treatments simply have a palliative effect on the relief of symptoms whereas not considering the essential problem of the cartilage disorder. Additionally, long-term consumption of these treatments has possible adverse events that might result drastic outcomes such as gastrointestinal problems, unwanted cardiovascular effects and adverse events on the cartilage^8^. Meanwhile, nutritional intervention demonstrates a continuing approach for management and inhibiting OA as an accompaniment to the traditional treatment of OA^9–12^. Nutraceutical supplements, such as chondroitin sulfate (CS), glucosamine sulfate (GS) and Methylsulfonylmethane (MSM), have been applied to manage OA and relieve symptoms in recent years^13^. Nutraceuticals are described as dietary supplements that comprise a condensed form of a considered bioactive ingredient, initially isolated from food, however existing in a nonfood matrix, and consumed to preserve or increase health situation in the amounts beyond those accessible from common foods^13^. Nevertheless, there is no agreement in regard to applying the term “nutraceutical” or “dietary supplement”. The “active aging” is a principle objective of dietary supplements, as indicated by the developing sales of vitamins and minerals^14^. Dietary bioactive combinations have been revealed to be impressive in the improvement of clinical symptoms and in decreasing inflammatory indices in subjects with OA^15^. Presently 69% of subjects with OA receive various forms of dietary supplements for their problem^16^.

Even though there are several publications in the medical literature in regard to the use of nutraceuticals as a complementary treatment of OA, there have been variable findings concerning whether or not these nutrients have any beneficial consequence. The purpose of this study is to perform a systematic review and meta-analysis of relevant randomized controlled trials (RCTs) to assess the efficiency of different dietary supplements in the management of the symptoms of hip/knee OA.

## Methods

The primary purpose of this systematic review and meta-analysis was to evaluate the efficacy and safety of dietary supplements in subjects with knee or hip OA. The current study has been planned based on the instructions in the Cochrane Collaboration handbook and Preferred Reporting Items for Systematic Reviews and Meta-Analyses (PRISMA) statement. The study question was framed according to the PICOS (participants, interventions, comparators, outcomes, study design) criteria (Table 1), is as follows: Do nutraceutical supplements influence pain and functional status in patients with hip/knee osteoarthritis?

**Table 1:** PICOS criteria for inclusion and exclusion of studies.

Parameter	Description
Population	Adult participants who have been diagnosed with hip or knee OA
Intervention	Nutraceutical (including dietary supplements, herbal food or medicinal food) administered for ≥ 2 weeks
Comparator	Any comparator
Outcomes	Outcomes regarding at least one of the following indices: WOMAC total, WOMAC pain, WOMAC stiffness, WOMAC physical function, VAS
Study design	Randomized controlled clinical trial with a crossover or parallel design

### Literature search

Several search strategies were employed to recognize eligible studies. A medical librarian (FB) in an argument with the team (DA, ND and FB) performed a precise and comprehensive academic literature search of the titles, abstracts and keywords of all studies for competency independently through electronic databases (MEDLINE, Web of Science, Cochrane Library, Scopus, EMBASE, Google Scholar, Clininaltrial.gov, Science direct, and ProQuest in addition to SID, Magiran, Irandoc, and Iranmedex for Persian language literature) up to January 2020. Duplicate studies were excluded. At the same time, a hand search of the related references and cited articles of the included studies was conducted to recognize other appropriate studies that were lost by electronic search.

Search terms included a mix of Medical Subject Headings (MeSH) and a literature search was performed using the following MeSH terms for key concepts (with assistance from a librarian) targeting dietary supplements and hip or knee OA such as : (“supplement ”(All Fields) OR “nutraceuticals”(All Fields) OR “vitamin”(All Fields) OR “mineral”(All Fields) OR “plant”(All Fields)) AND (“OA” OR “osteoarthritis”(All Fields) OR “knee osteoarthritis”(All Fields) OR “hip osteoarthritis”(All Fields) OR “knee OA”(All Fields) OR “hip OA”(All Fields)). After the primary search, titles and abstracts were sent out from EndNote X7 into Microsoft Excel to be screened. Three reviewers separately reviewed all titles and abstracts and full texts (DA, ND, and MH). A fourth reviewer was conferred if discrepancies happened.

### Inclusion and exclusion criteria

Inclusion criteria to choose studies for this systematic review and meta-analysis were: (1) RCT (either parallel or crossover designs); (2) a nutraceutical as an intervention either as an adjunctive to standard medicine or as a monotherapy and (3) adults who have been diagnosed with hip or knee OA; (4) sufficient data reported about mean changes for Western Ontario and McMaster Universities Arthritis (WOMAC) index (total score and subscales) and/or Visual analogue scale (VAS) at baseline and at the end of the trial in both intervention and placebo/control groups. Then selected possible clinical trials were excluded based on the exclusion criteria as follows: (1) duplications; (2) subjects have other critical diseases such cardiovascular disease, cancer, diabetes, etc.; (3) Studies with a short period of follow‐up (< 2 weeks); (4) review articles, semi-experimental studies without a control arm, animal studies, study protocols, letter to editors, case reports, case series, observational studies (cross-sectional, case–control and cohort) and unpublished trials.

No language limitations were applied to the search, but only studies published in English or Persian were incorporated because of translation constraints. Trials without full text and those that couldn’t attain the minimum quality appraisement score were not included in this systematic review.

### Quality and risk-of-bias assessment

To estimate the risk of systematic errors in the all involved clinical trials, two authors (ND and FB) individually evaluated the risk of bias according to the Cochrane Collaboration consists of the subsequent domains: “randomization sequence generation, allocation concealment, blinding of subjects, personal, and outcome assessment, incomplete outcome data, and selective outcome reporting, as well as other sources of bias”. Incompatibilities between reviewers, were resolved by the fourth author (MH). All studies were judged for each series of bias separately, and the studies were decided to take a score of bias as “low risk”, “high risk”, or “unclear risk” if data was inadequate.

### Data extraction

One reviewer extracted the data and abstracted it into an electronic form designed for this review, and a second reviewer confirmed it. Information extracted included: the first author’s name, publication details, location of the study, inclusion and exclusion criteria; the number of subjects for intervention and placebo groups, type of intervention, study design and duration, the mean and standard deviation (SD) for VAS and WOMAC index at baseline and at the end of the intervention in both intervention and control groups and safety.

### The outcome measures

The studies that met inclusion criteria were reviewed and the outcomes of these RCTs that could be retained for meta-analysis were considered as the primary outcome in this review. Thereupon, the primary outcome measures included for this review were mean changes in WOMAC total, WOMAC pain, WOMAC stiffness, WOMAC physical function and pain (VAS).

### Data synthesis and analysis

The number of subjects in each intervention group with mean and SD of study outcomes before and after the intervention was extracted from the articles included in the study. Then, the mean difference of study outcome was calculated and the mean difference of study outcomes was compared between the two groups. Because of the different scales used in the articles included in the study for the WOMAC index and VAS, the standardized mean difference (SMD) of the outcome changes between the two groups was considered as the effect size in this study. The random effects model was used to combine the effect sizes calculated in the articles. Heterogeneity between studies was assessed by Cochran's (Q) and I^2^ statistics, which expressed the percentage of variations between studies. In case of high heterogeneity between included studies, we performed subgroup analysis according to the treatment duration (< 10 months, 10–20 months and > 20 months) to evaluate the impression of these factors on the results. The Meta package in R software was used for data analysis. A p-value less than 0.05 was considered as significant level.

### Publication bias

Egger's Regression Test and Funnel Plot were used to evaluate the presence or absence of publication bias. Publication bias was assessed for each study outcome. The Trim and Fill method was used to investigate the effect of publication bias on the results of the study.

## Results

### Study selection process

**Figure 1:**
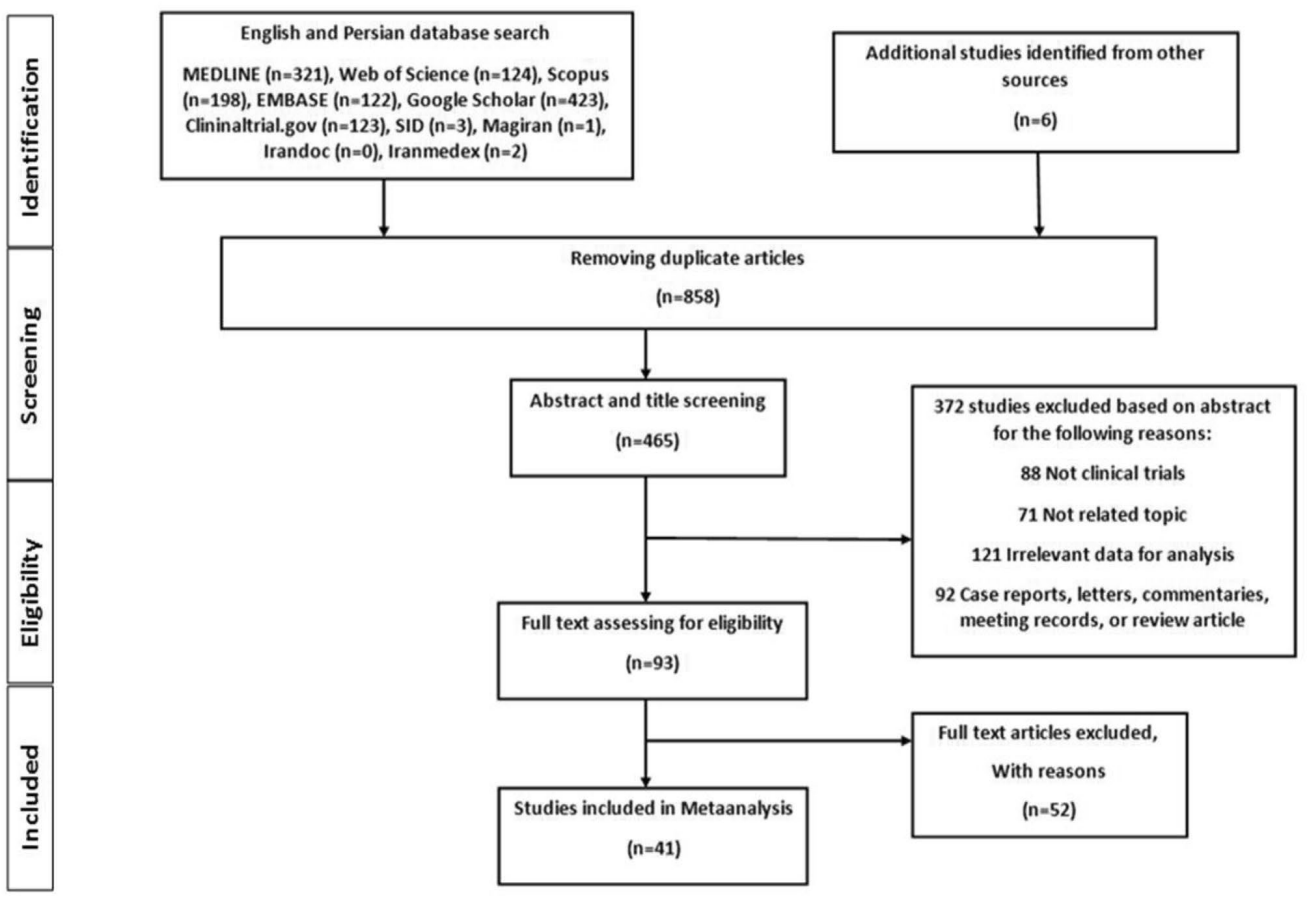
PRISMA diagram for the search and selection process of articles considered in this review.

The systematic searching of the databases identified 1323 articles, of which 858 were excluded as duplicates, 372 were excluded by title and abstract and 52 were excluded after reviewing full texts (Fig. 1).

### Study characteristics

This comprehensive systematic review and meta-analysis including 42 RCTs (4160 participants) and 33 supplements assessed the clinical effectiveness of different nutraceutical supplementation in the management of knee/hip OA symptoms, principally concentrating on pain and functional outcomes. The included articles in this systematic review were full articles published from January 2000 to March 2020. Papers were written in English or Persian. The details of the studies are summarized in Table 2.

**Table 2:** Summary table of included studies evaluating the effect of nutraceutical supplements in osteoarthritis.

Author (year)	Location	Inclusion criteria	Sample size and treatment (dosage)	Sample size at the end of treatment	Concomitant treatment	Design and study duration	Main outcomes
Reginster 2001^17^	Belgium	Knee OA(mild to moderate severity according to KLS) Age ≥ 50 years	1. GS (n = 106) (1500 mg/day) 2. Placebo (n = 106)	1. GS (n = 68) (1500 mg/day) 2. Placebo (n = 71)	Symptomatic treatments [Paracetamol 500 mg OR one NSAIDs (diclofenac 50 mg OR piroxicam 20 mg OR proglumetacin 150 mg)]	RCT 3 years	JSW, WOMAC index (total, pain, stiffness and physical function)
Appelboom 2001^18^	Belgium	Knee OA (unknown severity) Age: 45–80 years VAS ≥ 30 mm Lequesne index : 4–12	1. ASU (300 mg × 1/day) (n = 86) 2. ASU (600 mg × 1/day) (n = 86) 3.Placebo (n = 88)	1. ASU (300 mg × 1/day) (n = 74) 2. ASU (600 mg × 1/day) (n = 75) 3.Placebo (n = 76)	Symptomatic treatments (NSAIDs and analgesics)	RCT 3 months	Pain (VAS), LI
Jung 2001^19^	Korea	Knee OA (unknown severity) Age: 35–75 years VAS ≥ 35 mm	1. SKI 306X (mixture of Clematis mandshurica, Trichosanthes kirilowii and Prunella vulgaris) (200 mg × 3/day) (n = 24) 2. SKI 306X (400 mg × 3/day) (n = 24) 3. SKI 306X (600 mg × 3/day) (n = 24) 4. Placebo(n = 24)	1. SKI 306X (200 mg × 3/day) (n = 24) 2. SKI 306X (400 mg × 3/day) (n = 23) 3/ SKI 306X (600 mg × 3/day) (n = 23) 4. Placebo(n = 23)	–	RCT 4 weeks	Pain (VAS), LI
Schmid 2001^20^	Germany	Hip or knee OA (unknown severity) Age > 18 years (men) or > 50 years (women)	1. Willow bark extract (240 mg × 1/day) (n = 39) 2. Placebo (n = 39)	1. Willow bark extract (240 mg × 1/day) (n = 39) 2. Placebo (n = 39)	–	RCT 2 weeks	WOMAC (pain, stiffness and physical function), Pain (VAS)
Colker 2002^21^	USA	Knee OA (unknown severity) Age ≥ 35 years	1. Micronutrient-containing beverage (12 oz/day) (n = 20) 2. Placebo (n = 20)	1. Micronutrient-containing beverage (12 oz/day) (n = 16) 2. Placebo (n = 15)	–	RCT 6 wk	Modified KOOS, WOMAC, Pain (VAS)
Zenk 2002^22^	USA	OA (unknown severity) Age > 19 years	1. MPC (2000 mg × 2/day) 2. GS (500 mg × 3/day) 3. Placebo (n = 42)	1. MPC (2000 mg × 2/day) (n = 12) 2. GS (500 mg × 3/day) (n = 13) 3. Placebo (n = 10)	Symptomatic treatments (Naproxen 220 mg, ibuprofen 200 mg, acetaminophen 325 mg, and acetylsalicylic acid 325 mg)	RCT 6 weeks	WOMAC (total, pain, stiffness and physical function)
Lequense 2002^23^	France	Hip OA (mild to moderate severity according to KLS) Age: 50–80 years	1. ASU (300 mg × 1/day) (n = 85) 2. Placebo (n = 78)	1. ASU (300 mg × 1/day) (n = 45) 2. Placebo (n = 51)	Symptomatic treatments [NSAIDs (diclofenac, flurbiprofen, ibuprofen, indomethacin, ketoprofen, paroxen, piroxicam, tenoxicam)] AND/OR analgesics	RCT 2 years	JSW, LI, Pain (VAS)
McAlindon 2004^24^	England	Knee OA (mild to severe severity according to KLS) Age ≥ 45 years	1. GS (1.5 g/d) (n = 101) 2. Placebo (n = 104)	1. GS (1.5 g/d) (n = 93) 2. Placebo (n = 93)	Symptomatic treatments (Acetaminophen)	RCT 12-week,	WOMAC (total, pain, stiffness and physical function)
Miller 2005^25^	India	Knee OA(mild to moderate severity according to KLS) Age ≥ 20 years VAS ≥ 50 mm	1. Sierrasil (containing silicate minerals of calcium, magnesium, potassium, sodium and aluminum, among others) (n = 25) (3 g/day) 2. Sierrasil (n = 24) (2 g/day) 3. sierrasil (2 g/day) + cat's claw extract (100 mg/ day) (n = 29) 4. Placebo (n = 29)	1. Sierrasil (n = 20) (3 g/day) 2. Sierrasil (n = 22) (2 g/day) 3. sierrasil (2 g/day) + cat's claw extract (100 mg/ day) (n = 26) 4. Placebo (n = 23)	Symptomatic treatments (Acetaminophen up to 2 g/day)	RCT 8 weeks	WOMAC (total, pain, stiffness and physical function)
Kim 2006^26^	USA	Knee OA (mild to moderate severity according to KLS) Age > 40 years VAS > 40 mm global assessment (GA) > 2	1. MSM (1 g × 2/day for 3 days, 2 g × 2/day for 4 days, then 3 × 2 g/day) (n = 25) 2. Placebo (n = 25)	1. MSM (1 g × 2/day for 3 days, 2 g × 2/day for 4 days, then 3 × 2 g/day) (n = 21) 2. Placebo (n = 19)	Symptomatic treatments (Acetaminophen up to 2.6 g/day)	RCT 12-week	Pain (VAS), WOMAC (total, pain, stiffness and physical function)
Pavelka 2007^27^	Czech Republic and Slovak Republic	Knee OA (mild to moderate severity according to KLS) Age: 40–75 years VAS ≥ 40 mm WOMAC pain ≥ 2	1.Diacerein (50 mg × 1/day) (n = 84) 2. Placebo (n = 84)	1.Diacerein (50 mg × 1/day) (n = 76) 2. Placebo (n = 76)	Symptomatic treatments (Acetaminophen up to 1500 mg/day)	RCT 3 months	WOMAC (total, pain, stiffness and physical function)
Farid 2007^28^	Iran	Knee OA (mild severity according to ACR) Age: 25- 65 years WOMAC ≥ 40 Pain ≥ 50% of the time in last 3 months	1. Pycnogenol (n = 19) (150 mg × 1/day) 2. Placebo (n = 18)	1. Pycnogenol (n = 18) (150 mg × 1/day) 2. Placebo (n = 17)	Symptomatic treatments (NSAIDs and COX-2 inhibitors)	RCT 90 days	WOMAC (total, pain, stiffness and physical function)
Mehta 2007^29^	India	Knee OA (mild to moderate severity according to KLS) VAS: ≥ 40 mm and ≤ 80 mm Age ≥ 20 years	1. GS (750 mg × 2/day) (n = 47) 2.Reparagen (blend of vincaria: an extract of Uncaria guianensis (300 mg) and RNI 249: an extract of Lepidium meyenii (1500 mg)) (900 mg × 2/day) (n = 48)	1. GS (750 mg × 2/day) (n = 41) 2.Reparagen (900 mg × 2/day) (n = 38)	Symptomatic treatments (Acetaminophen up to 1500 mg/day for the first 4 weeks and 1000 mg/day for the last 4 weeks)	RCT 8 weeks	WOMAC (total, pain, stiffness and physical function), Pain (VAS)
Alishiri GH.H.2007^30^	Iran	Knee OA (mild severity according to KLS) Age: 50–80 years VAS: ≥ 40 mm	1. Elaeagnus Angustifolia extract (100 mg × 2/day) (n = 40) 2.Acetaminophen (500 mg × 2/day) (n = 40) 3. Placebo(n = 40)	1. Elaeagnus Angustifolia extract (100 mg × 2/day) (n = 38) 2.Acetaminophen (500 mg × 2/day) (n = 37) 3. Placebo(n = 40)	–	RCT 7 weeks	Pain (VAS), LI
Sengupta 2008^8^	India	Knee OA (mild to moderate symptoms) Age: 40–80 years VAS: 40–70 mm LF Index score > 7 Ability to walk	1.5-Loxin (Boswellia serrata extract contain at least 30 percent 3-O-Acetyl-11-keto-β-boswellic acid) (250 mg × 1/day) (n = 25) 2. 5-Loxin (100 mg × 1/day) (n = 25) 3.Placebo (n = 25)	1.5-Loxin (250 mg × 1/day) (n = 23) 2.5- Loxin (100 mg × 1/day) (n = 24) 3.Placebo (n = 23)	Symptomatic treatments (ibuprophen up to 1,200 mg/day)	RCT 90-day	Pain (VAS), LI, WOMAC (pain, stiffness and physical function)
Kalman 2008^31^	United States	Knee OA (mild to severe severity according to KLS) Age ≥ 40 years	1. Chicken comb extract (80 mg × 1/day) (n = 11) 2. Placebo (n = 9)	1. Chicken comb extract (80 mg × 1/day) (n = 8) 2. Placebo (n = 8)	Symptomatic treatments (paracetamol up to 2000 mg/daY)	RCT 8 weeks	WOMAC (total, pain, stiffness and physical function), QOL (SF-36)
Frestedt 2008 ^32^	USA	Knee OA (moderate to severe severity according to ACR) Age: 25–75 years WOMAC total ≤ 75	1. Aquamin (2400 mg × 1/day) (n = 20) 2.Glucosamine sulfate (1500 mg × 1/d) (n = 19) 3. Glucosamine sulfate (1500 mg × 1/day) + Aquamin (2400 mg × 1/day) (n = 15) 4.Placebo (n = 16)	1. Aquamin (2400 mg × 1/day) (n = 15) 2.Glucosamine sulfate (1500 mg × 1/d) (n = 14) 3. Glucosamine sulfate (1500 mg × 1/day) + Aquamin (2400 mg × 1/day) (n = 12) 4.Placebo (n = 9)	Symptomatic treatments (Acetaminophen, 325 mg, 1–2 tablets every 4–6 h)	RCT 12 weeks	WOMAC (total, pain, stiffness and physical function), 6 MWD
Jacquet 2009^33^	France	Knee or hip (unknown severity) Age: 40–80 years	1. Phytalgic (fish-oil, vitamin E, *Urtica dioica*) (n = 41) 2. Placebo (n = 40)	1. Phytalgic (fish-oil, vitamin E, *Urtica dioica*) (n = 40) 2. Placebo (n = 36)	Symptomatic treatments (analgesics and/or NSAIDs)	RCT 3 months	WOMAC (total, pain, stiffness and physical function)
Frestedt 2009^34^	USA	Knee OA (moderate to severe severity according to ACR) Age: 35–75 years WOMAC total ≤ 75	1.Aquamin (A calcium and magnesium-rich seaweed-derived multi-mineral supplement) (801 mg × 3/day) (n = 8) 2.Placebo (n = 14)	1 .Aquamin (801 mg × 3/day) (n = 5) 2.Placebo (n = 9)	Symptomatic treatments (NSAIDs)	Pilot RCT 12 weeks	6 MWD, ROM WOMAC (total, pain, stiffness and physical function)
Ruff 2009^35^	USA	Knee OA (mild to severe severity according to ACR) Age ≥ 18 years VAS ≥ 30 mm	1. NEM (500 mg × 1/d) (n = 29) 2. Placebo (n = 31)	1. NEM (500 mg × 1/d) (n = 20) 2. Placebo (n = 18)	Symptomatic treatments (Acetaminophen)	RCT 8 weeks	WOMAC (total, pain, stiffness and physical function) Pain (VAS)
Farid 2010^36^	Iran	Knee OA (mild to severe severity according to ACR) Age: 25–65 years WOMAC pain subscale index ≥ 40	1. PFP (150 mg × 1/d) (n = 20) 2. Placebo (n = 20)	1. PFP (150 mg × 1/d) (n = 17) 2. Placebo (n = 16)	Symptomatic treatments (NSAIDs and COX-2 inhibitor)	RCT 2 months	WOMAC (total, pain, stiffness and physical function)
Sengupta 2010^37^	India	Knee OA(unknown severity) Age: 40–80 years VAS: 40–70 mm LF Index > 7 Ability to walk	1. 5-Loxin (100 mg × 1/day) (n = 20) 2. 100 mg of Aflapin (Boswellia serrata extract) (100 mg × 1/day) (n = 20) 3. Placebo (n = 20)	1. 5 -Loxin (100 mg × 1/day) (n = 19) 2. 100 mg of Aflapin (100 mg × 1/day) (n = 19) 3. Placebo (n = 19)	Symptomatic treatments (ibuprofen up to 1200 mg/day)	RCT 90-day	Pain (VAS), LI, WOMAC (pain, stiffness and physical function)
Debbi 2011^38^	Israel	Knee OA (unknown severity) Age: 45–90 years	1. MSM (1.125 g × 3/day) (n = 25) 2. Placebo (n = 25)	1. MSM (1.125 g × 3/day) (n = 25) 2. Placebo (n = 25)	Unknown	RCT 12 weeks	WOMAC (total, pain, stiffness, physical function), Pain (VAS), QOL (SF-36), KSKS, KSFS
Notarnicola 2011^39^	Italy	Knee OA (moderate severity according to KLS) Age: > 45 and < 85 years VAS ≥ 2 cm on a 10 cm LI > 2	1. MSM 5 gr and 7.2 mg of titred Boswellic Acids (n = 30) 2. Placebo (n = 30)	1. MSM 5 gr and 7.2 mg of titred Boswellic Acids (n = 30) 2. Placebo (n = 30)	Symptomatic treatments (paracetamol 500 mg) OR NSAIDs (pyroxicam 20 mg, diclofenac 50 mg)/day	RCT 60 days	Pain (VAS), LI
Schauss 2012^40^	United States	Knee and/or hip OA (unknown severity) Age: 40–70 years VAS ≥ 4	1. BioCell Collagen (500 mg × 4/day) (n = 40) 2. Placebo (n = 40)	1. BioCell Collagen (500 mg × 4/day) (n = 35) 2. Placebo (n = 33)	Symptomatic treatments ( Paracetamol up to 4 gr/day)	RCT 70 days	Pain (VAS), WOMAC (total, pain, stiffness and physical function)
McAlindon 2013^41^	United States	Age ≥ 45 years (mild to severe severity according to KLS) Knee OA	1.Cholecalciferol (initial dose 2000 IU/day)(n = 73) 2. Placebo (n = 73)	1.Cholecalciferol (initial dose 2000 IU/day)(n = 64) 2.Placebo (n = 60)	Conventional treatments ( Acetaminophen & NSAIDs	RCT 2 years	WOMAC (pain and function )
Ebrahimi 2014^42^	Iran	Knee OA (mild to moderate severity according to KLS) Sex: female Age: 40–70 years BMI: 25–34.9 kg/m^2^	1. Whole fruit powder of *Elaeagnus angustifolia* L. (n = 30) (15 g × 1/day) 2. Medulla powder of *Elaeagnus angustifolia* L. (n = 30) (15 g × 1/day) 3. Placebo (n = 30)	1. Whole fruit powder of *Elaeagnus angustifolia* L. (n = 26) (15 g × 1/day) 2. Medulla powder of *Elaeagnus angustifolia* L. (n = 27) (15 g × 1/day) 3. Placebo (n = 25)	Conventional treatments ( Acetaminophen & NSAIDs (Celecoxib, Ibuprofen, Naproxen)	RCT 8 weeks	WOMAC (total, pain, stiffness and physical function)
Kolahi 2015^43^	Iran	Knee OA (mild to moderate severity according to KLS) Age: 40 to 60 years Sex: female BMI: 25–34.9 kg/m^2^	1. L-carnitine ( 250 mg × 3/day) (n = 36) 2. Placebo (n = 36)	1. L-carnitine (250 mg × 3/day) (n = 33) 2. Placebo (n = 36)	Symptomatic treatments (Acetaminophen)	RCT 8 weeks	WOMAC (total, pain, stiffness and physical function)
Kumar 2015^44^	India	Knee OA (mild to severe severity according to KLS) Age: 30–65 years	1. PCP daily twice (5 g dissolved in 250 mL of milk or water) (n = 20) 2. Placebo (n = 10)	1. PCP daily twice (5 g dissolved in 250 mL of milk or water) (n = 19) 2. Placebo (n = 11)	Symptomatic treatments (Aceclofenac sodium 100 mg/day)	RCT 13 weeks	WOMAC, Pain (VAS), QOL
Dehghan 2015^45^	Iran	Knee OA (mild to moderate severity according to the Ahlback classification) VAS ≥ 4 cm Age: 30–60 years	1. Vitamin B Complex (× 2/day) (n = 40) 2. Placebo (n = 40)	1. Vitamin B Complex (× 2/day) (n = 38) 2. Placebo (n = 35)	Symptomatic treatments (Diclofenac 100 mg /day)	RCT 21 days	Pain (VAS), WOMAC (pain, stiffness and physical function)
Jin 2016^46^	Australia	Knee OA (mild to moderate severity according to the Altman and Gold atlas) Age: 50–79 years old VAS ≥ 20 mm Serum vitamin D level: > 12.5 and < 60 nmol/L	1. Vitamin D3 (50,000 IU × 1/month)(n = 209) 2. Placebo (n = 204)	1. Vitamin D3 (50,000 IU × 1/month)(n = 209) 2. Placebo (n = 204)	Unknown	RCT 24 months	WOMAC (total, pain, stiffness and physical function), Pain (VAS)
Stebbings 2016^47^	New Zealand	Knee or hip OA (unknown severity) Age: 35–75 years BMI < 40 kg/m^2^ VAS ≥ 30 mm on a 100-mm	1. ART (150 mg × 1/day) (n = 14) 2 ART high dose (300 mg × 1/day) (n = 14) 3. Placebo (n = 14)	1. ART (150 mg × 1/day) (n = 12) 2 ART high dose (300 mg × 1/day) (n = 9) 3. Placebo (n = 13)	Symptomatic treatments (NSAIDs and analgesics)	RCT 12 weeks	WOMAC (total, pain, stiffness and physical function), Pain (VAS)
Lugo 2016^48^	India	Knee OA (mild severity according to KLS) Age: 40–75 years BMI: 18–30 kg/m^2^ LI score: 6–10 VAS score: 40–70 mm	1. UC- II (40 mg × 1/day) (n = 63) 2. GS (1500 mg × 1/day) + CS (1200 mg × 1/day) (n = 65) 3. Placebo(n = 58)	1. UC- II (40 mg × 1/day) (n = 54) 2. GS (1500 mg × 1/day) + CS (1200 mg × 1/day) (n = 57) 3. Placebo(n = 53)	Symptomatic treatments (Acetaminophen 1000 mg daily)	RCT 180-day	WOMAC (total, pain, stiffness and physical function), LI, Pain (VAS), ROM
Lubis 2017^49^	Indonesia	Knee OA (mild severity according to KLS)	1. GS (1500 mg × 1/day) + CS (1200 mg × 1/day) + saccharumlactis (500 mg × 1/day) (n = 49) 2. GS (1500 mg × 1/day) + CS (1200 mg × 1/day) + MSM (500 mg × 1/day) (n = 50) 3. Placebo (n = 48)	1. GS (1500 mg × 1/day) + CS (1200 mg × 1/day) + saccharumlactis (500 mg × 1/day) (n = 49) 2. GS (1500 mg × 1/day) + CS (1200 mg × 1/day) + MSM (500 mg × 1/day) (n = 50) 3. Placebo (n = 48)	Unknown	RCT 3 months	WOMAC, Pain (VAS)
Rafarf 2017^50^	Iran	Knee OA (mild severity according to KLS) Age: 38–60 years old Sex: female BMI: between 30–35 kg/m^2^	1. Pomegranate peel extract (PPE) (1000 mg/day) (n = 33) 2. Placebo (n = 33)	1. Pomegranate peel extract (PPE) (1000 mg/day) (n = 30) 2. Placebo (n = 30)	Symptomatic treatments (Acetaminophen 1000 mg + Glucosamine 500 mg per day)	RCT 8 weeks	KOOS (Total and subscales), Pain (VAS)
Lei 2017^51^	*China*	Knee OA (mild severity according to KLS) Age < 80 years	1. Skimmed milk containing probiotic LcS (n = 230) 2. Placebo (plain skimmed milk) (n = 231)	1. Skimmed milk containing probiotic LcS (n = 215) 2. Placebo (plain skimmed milk) (n = 218)	Unknown	RCT 6 months	WOMAC (total, pain, stiffness and physical function), Pain (VAS)
Shin 2018^52^	New Zealand	Knee OA (moderate to severe severity according to KLS) Age ≥ 50 years WOMAC pain score ≥ 5.0	1. DBE (550 mg/day) (n = 30) 2. Placebo (n = 30)	1. DBE (550 mg/day) (n = 26) 2. Placebo (n = 24)	Symptomatic treatments (Acetaminophen 2000 mg daily not more than twice per week)	RCT 12 weeks	WOMAC (total, pain, stiffness and physical function), Pain (VAS)
Dehghani 2018^53^	Iran	Knee OA (mild severity according to KLS) Age: 50–75 years Sex: female BMI: 25–40 kg/m^2^)	1. Garlic tablets (1000 mg × 1/day) (n = 40) 2. Placebo (n = 40)	1. Garlic tablets (1000 mg × 1/day) (n = 39) 2. Placebo (n = 37)	–	RCT 12-week	Pain (VAS)
Salimzadeh 2018^54^	Iran	Knee OA (unknown severity) Age: 50–75 years Sex: female BMI: 25–40 kg/m2)	1. Garlic tablet (1000 mg × 1/day) (n = 39) 2. Placebo (n = 37)	1. Garlic tablet (1000 mg × 1/day) (n = 38) 2. Placebo (n = 34)	–	RCT 12 weeks	WOMAC (total, pain, stiffness and physical function), body composition (weight, WC, BMI, FFM, FM, VAT)
Hancke 2019^55^	India	Knee OA(mild severity according to KLS) Age: 40–70 BMI ≥ 25 and ≤ 29.9 kg/m^2^ WOMAC pain score: 10–16	1. ParActin (300 mg × 1/day) (n = 37) 2. ParActin (600 mg × 1/day) (n = 35) 3. Placebo (n = 36)	1. ParActin (300 mg × 1/day) (n = 35) 2. ParActin (600 mg × 1/day) (n = 33) 3. Placebo (n = 35)	–	RCT 12 week	WOMAC (total, pain, stiffness and physical function), QOL (SF-36), FACIT score
Majeed 2019^56^	India	Knee OA (mild to moderate severity according to KLS) Age: 35–75 years VAS score > 4 cm	1. Boswellin: (β‐boswellic acids 87.3 mg × 2/day) (n = 24) 2. Placebo (n = 24)	1. Boswellin: (β‐boswellic acids 87.3 mg × 2/day) (n = 22) 2. Placebo (n = 20)	–	RCT 120 days	WOMAC, 6 MW, Pain (VAS), QOL(European Quality of life‐5 Dimension, JSW
Rondanelli 2019^57^	Italy	Knee OA (mild to moderate according to KLS) Aged ≥ 55 years BMI: 25–30 kg/m^2^ VAS: 40–70 mm	1. CS (600 × 1/mg) (n = 30) 2. Placebo (n = 30)	1. CS (600 × 1/mg) (n = 30) 2. Placebo (n = 30)	–	Pilot RCT 12 weeks	WOMAC, Pain (VAS), TLKS scale, QOL (SF-36), Body Composition (Weight, BMI, FFM, FM, VAT)

*6 MW* 6 min walking test, *ACR* American College of Rheumatology Classification Criteria for Knee Osteoarthritis, *ART* Artemisia annua extract, *ASU* Avocado soybean unsaponifiable, *BMI* body mass index, *CS* chondroitin sulfate, *DBE* Deer bone extract, *FACIT* Functional Assessment of Chronic Illness Therapy, *FFM* free fat mass, *FM* fat mass, *GS* Glucosamine sulphate, *JSW* joint space width, *KLS* Kellgren and Lawrence scoring system for classification of knee OA, *KOOS* Knee Injury and Osteoarthritis Outcome Score, *KSFS* Function Score, *LcS* Lactobacillus casei Shirota, *KSKS*, Knee Society Clinical Rating System for Knee Score, *LI* Lequesne's Index, *MPC* milk protein concentrate, *MSM* Methylsulfonylmethane, *NEM* natural egg membrane, *NSAIDs* Non-steroidal anti-inflammatory drugs, *ParActin* A. paniculata purified extract, *PFP* extract of the skin of the passion fruit, *PCP* Collagen peptides isolated from pork skin, *QOL* quality of life, *ROM* range of motion, *TLKS* Tegner Lysholm Knee Scoring, *VAS* Visual analogue scale, *VAT* visceral adipose tissue, *WOMAC* Western Ontario and McMaster Universities Arthritis.

### Risk of bias in included studies

**Figure 2:**
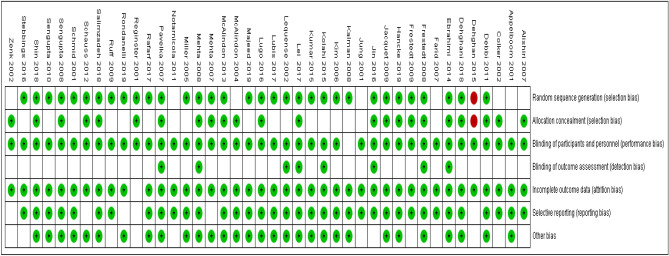
Diagram of bias in the included studies.

**Figure 3:**
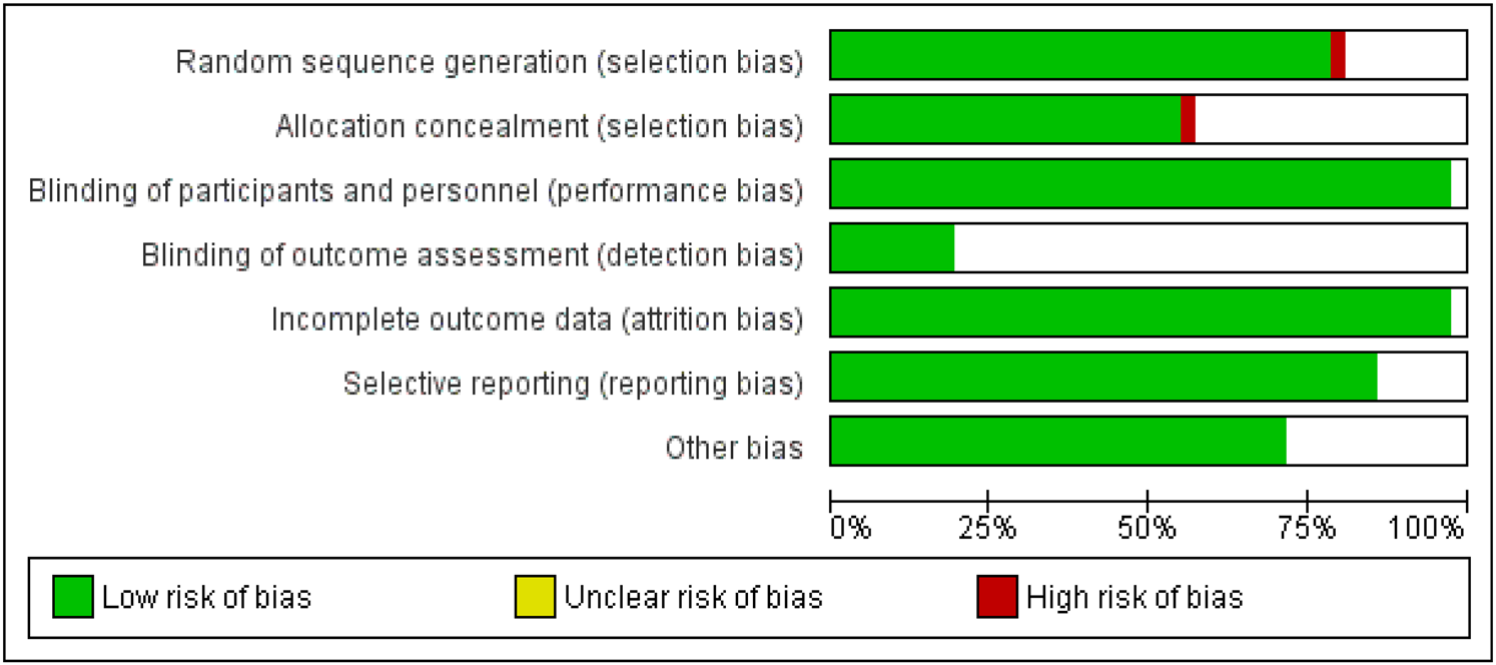
Diagram of bias in the included studies.

The methodological quality according to the researchers’ decisions on each risk of bias point for each included study is shown in Figs. 2 and 3.

### Efficacy of the intervention

#### WOMAC (total)

**Figure 4:**
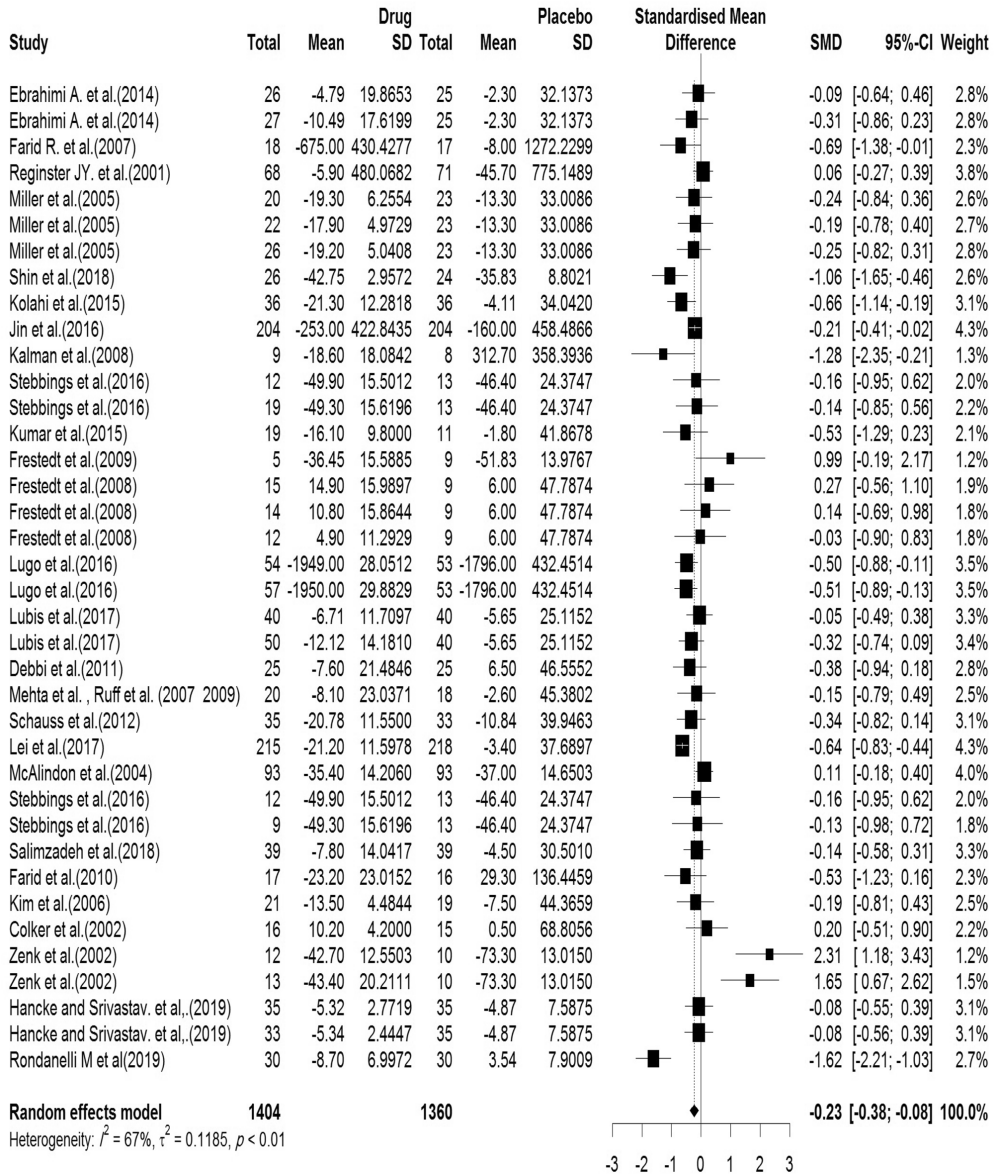
Forest plot presenting the standardized mean difference and 95% confidence interval for the impact of nutraceutical supplementation on WOMAC total score.

**Figure 5:**
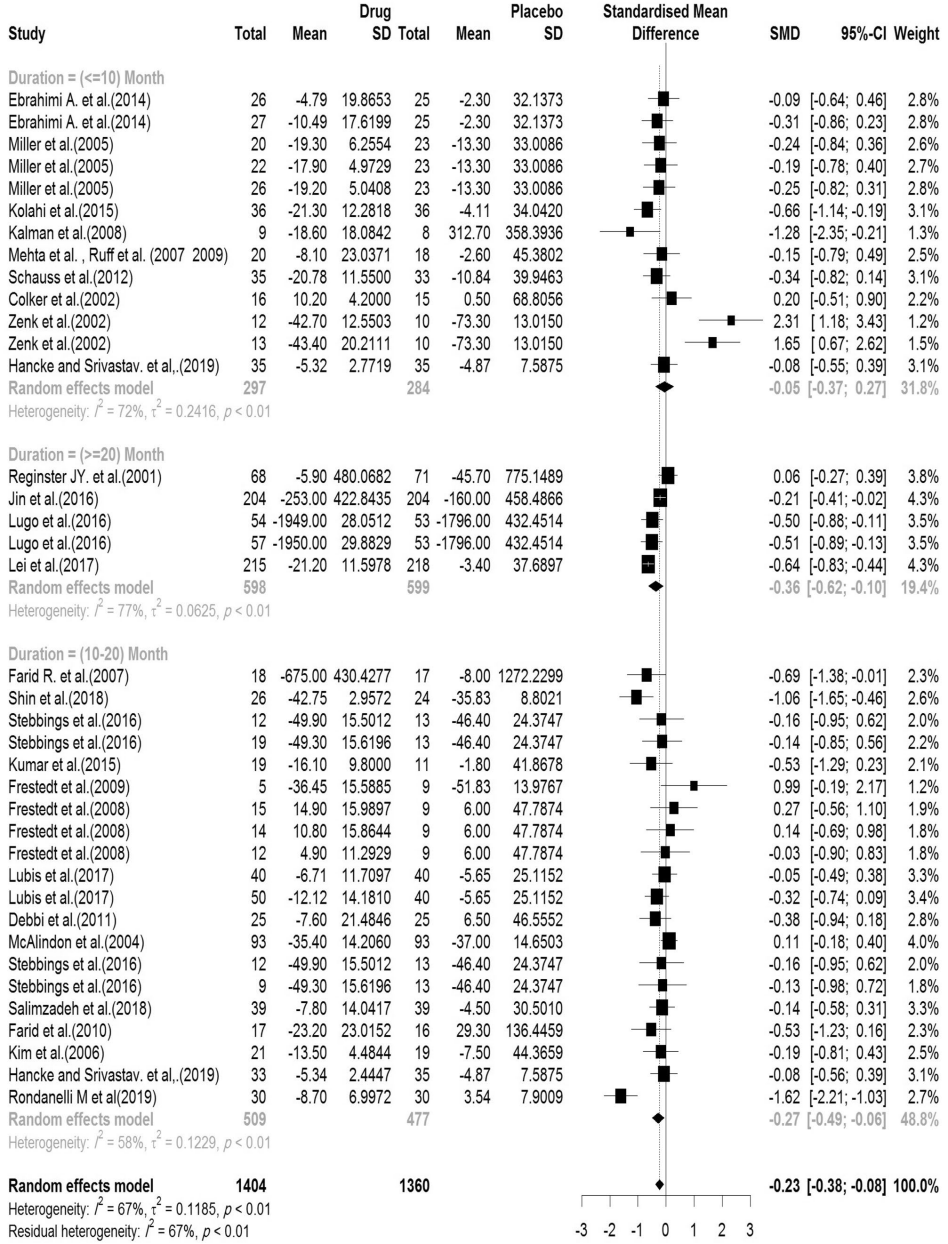
Forest plot presenting the impact of nutraceutical supplementation on WOMAC total score (subgroup analysis based on duration of supplementation).

The total score of the WOMAC was evaluated in the 28 articles reviewed. There were 1404 cases in the intervention group and 1360 in the control group. The mean follow-up duration of patients (lowest to maximum) was 17.4 (6–144) weeks. There was a significant heterogeneity between studies (Q-value = 110.58, df = 37, p-value < 0.001, I^2^ = 66.5%). Based on the meta-analysis results, it was observed that the Pooled Standardized Mean Difference between the intervention and control groups was 0.23 units (SMD = − 0.23, 95% CI − 0.37 to − 0.08, z-value = − 3.09, p-value = 0.002). Figure 4 shows the forest plot of the combination of results. Results of subgroup analysis according to the supplementation duration showed that the pooled effect size in studies with < 10 months as short term, 10–20 months as medium term and > 20 months as long term supplementation duration were 0.05, 0.27 and 0.36, respectively. Figure 5 shows the forest plot of the subgroups by the supplementation duration.

#### WOMAC (pain)

**Figure 6:**
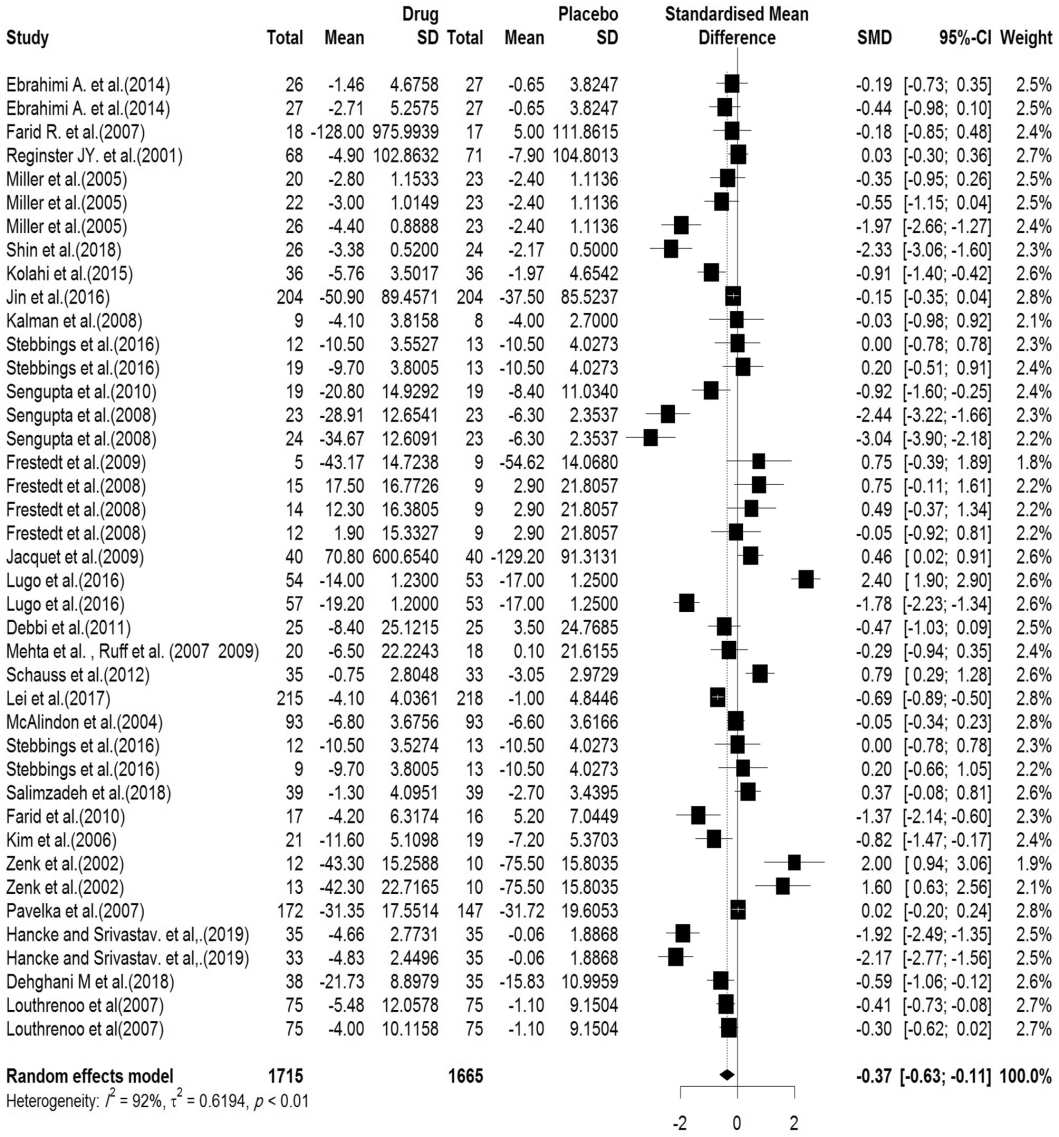
Forest plot presenting the standardized mean difference and 95% confidence interval for the impact of nutraceutical supplementation on WOMAC pain score.

**Figure 7:**
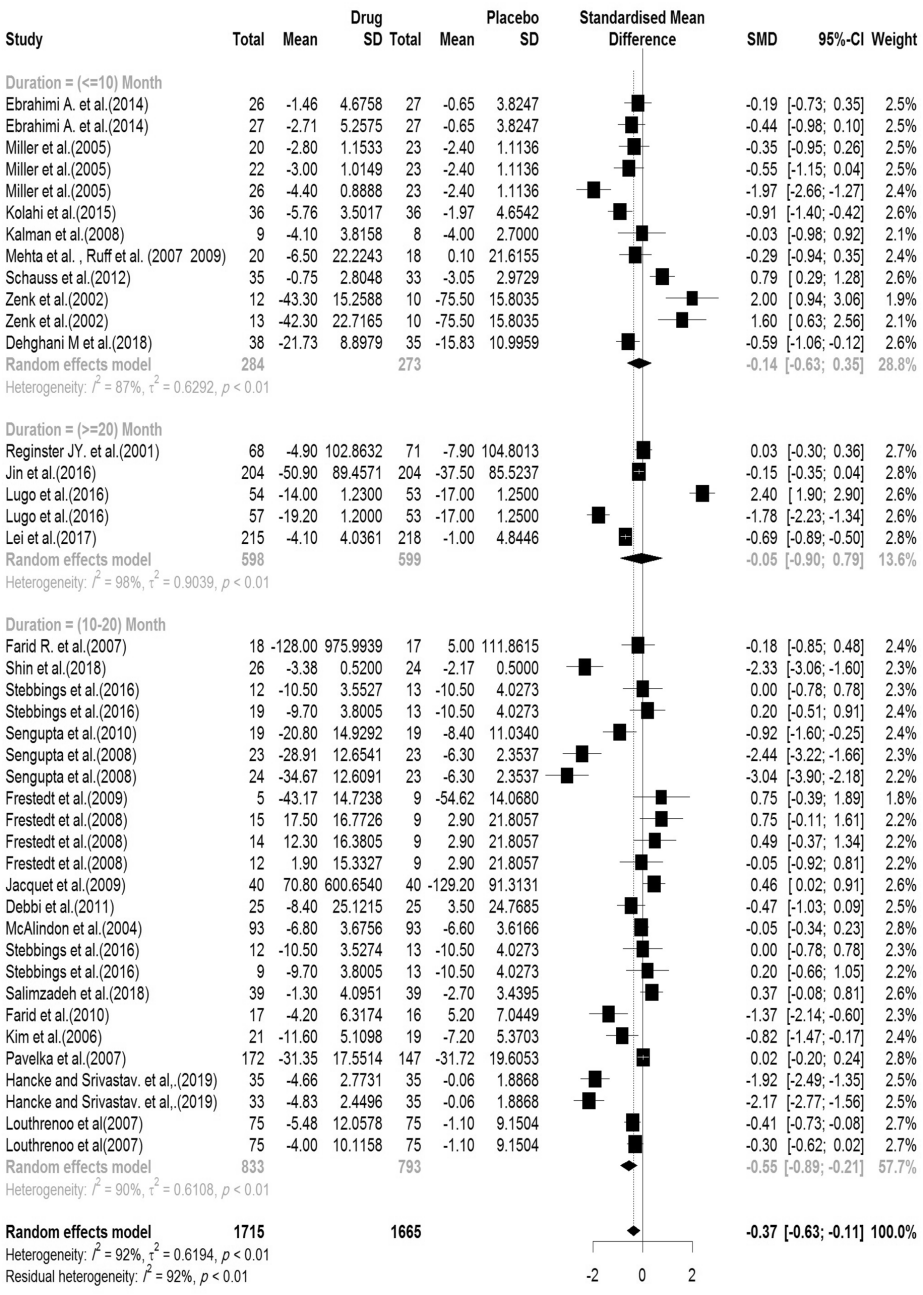
Forest plot presenting the impact of nutraceutical supplementation on WOMAC pain score (subgroup analysis based on duration of supplementation).

In the included articles, 30 articles evaluated the WOMAC pain subscale. There were 1715 subjects in the intervention group and 1665 subjects in the control group. The mean follow-up duration of patients (lowest to maximum) was 16.82 (3–144) weeks. There was a significant heterogeneity between studies (Q-value = 485.41, df = 40, p-value < 0.001, I^2^ = 92.2%). The Pooled Standardized Mean Difference between the intervention and control groups was 0.36 units (SMD = − 0.37, 95% CI − 0.63 to − 0.11, z-value = − 2.75, p-value = 0.006). The forest plot of the combination of results is presented in Fig. 6. The pooled effect size in studies with < 10 months as short term, 10–20 months as medium term and > 20 months as long term supplementation duration were 0.14, 0.55 and 0.05, respectively. The forest plot of the subgroups by the supplementation duration is presented in Fig. 7.

#### WOMAC (stiffness)

**Figure 8:**
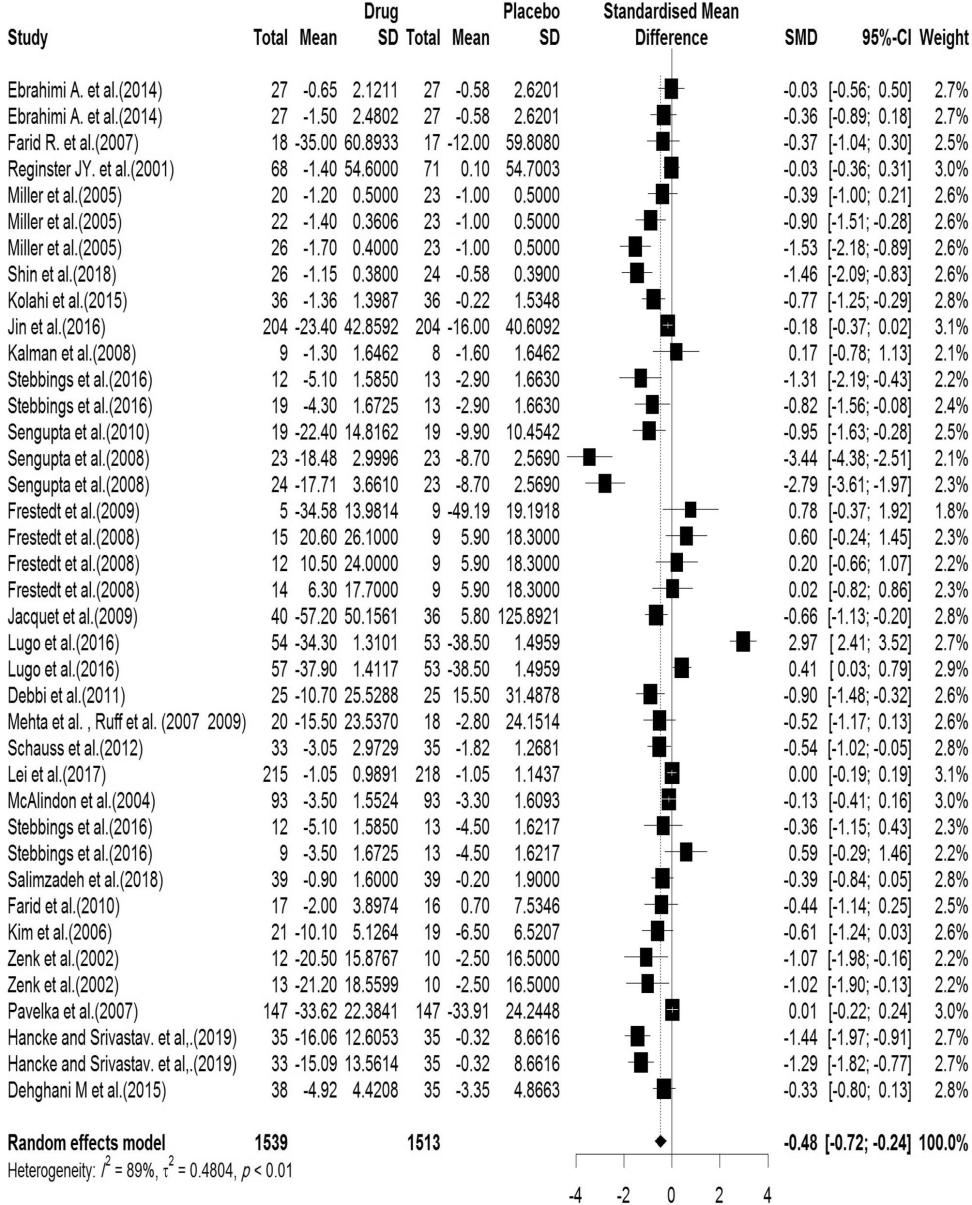
Forest plot presenting the standardized mean difference and 95% confidence interval for the impact of nutraceutical supplementation on WOMAC stiffness score.

**Figure 9:**
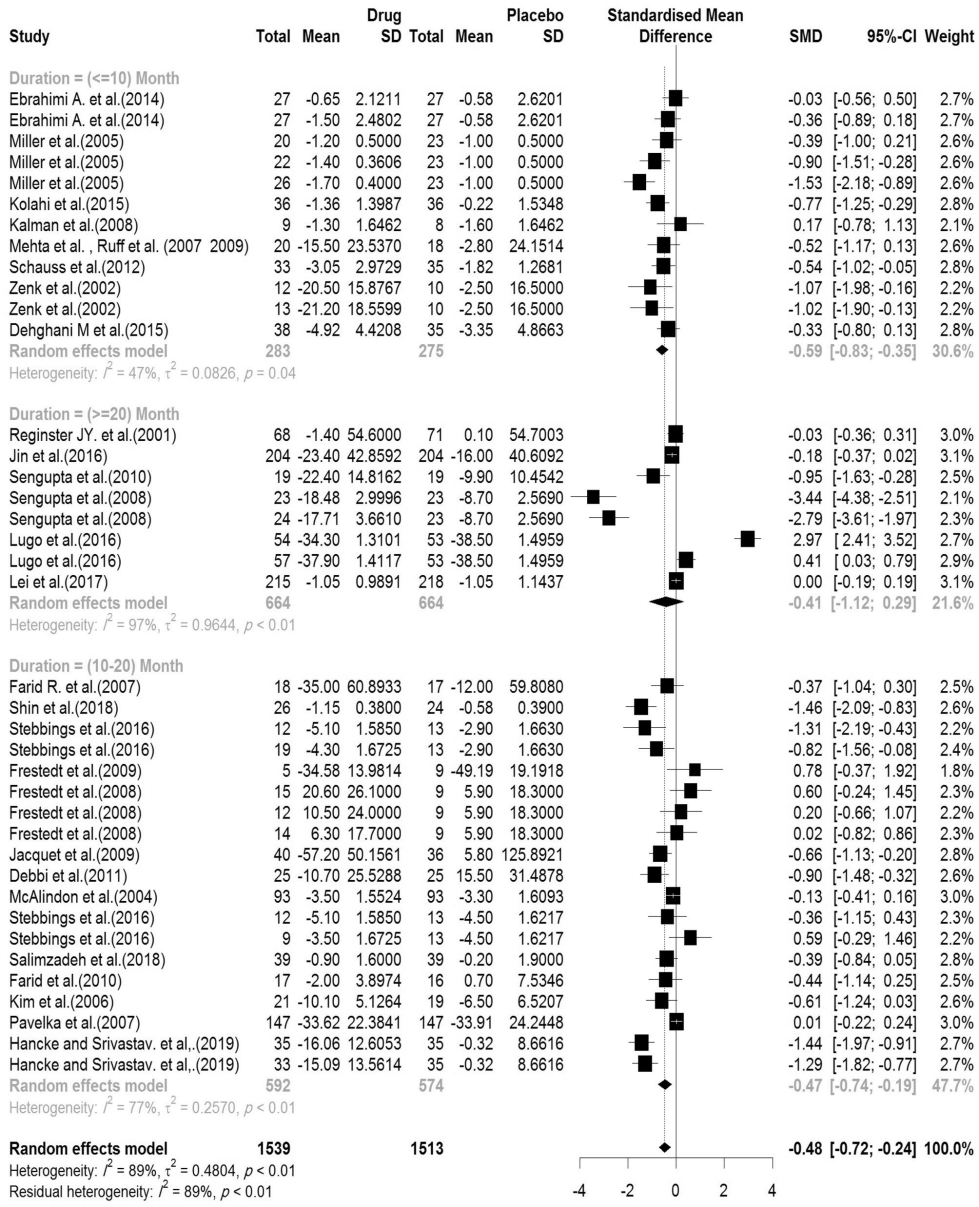
Forest plot presenting the impact of nutraceutical supplementation on WOMAC stiffness score (subgroup analysis based on duration of supplementation).

In the included articles, 29 articles assessed the WOMAC Stiffness subscale. There were 1539 subjects in the intervention group and 1513 subjects in the control group. The mean follow-up duration of patients (lowest to maximum) was 17.76 (3–144) weeks. There was a significant heterogeneity between studies (Q-value = 353.55, df = 38, p-value < 0.001, I^2^ = 88.8%). The Pooled Standardized Mean Difference between the intervention and control groups was 0.48 units (SMD = − 0.48, 95% CI − 0.72 to − 0.24, z-value = − 2.88, p-value < 0.001). The forest plot of the combination of results is presented in Fig. 8. The pooled effect size in studies with < 10 months as short term, 10–20 months as medium term and > 20 months as long term supplementation duration were 0.59, 0.47 and 0.41, respectively. The forest plot of the subgroups by the supplementation duration is presented in Fig. 9.

#### WOMAC (physical function)

**Figure 10:**
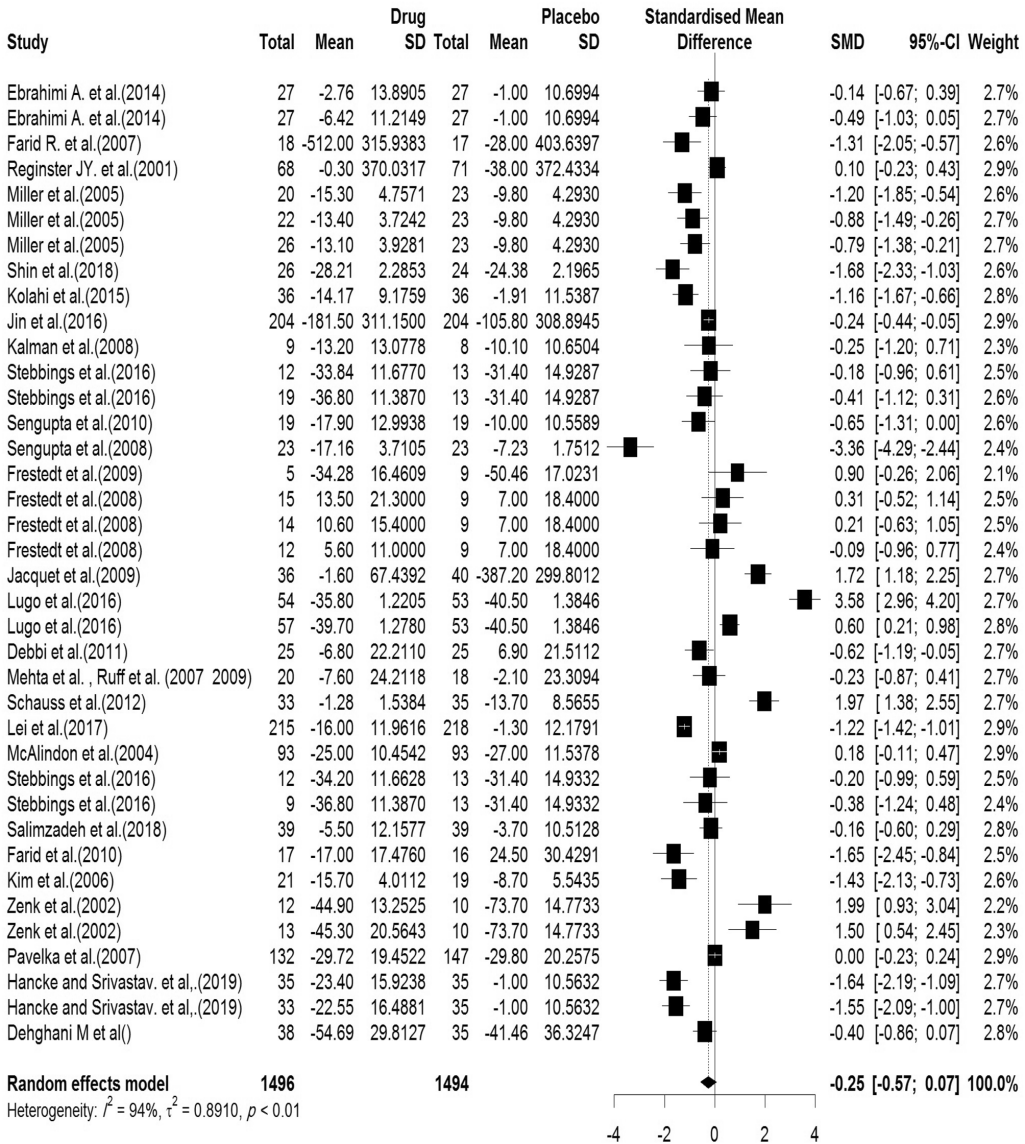
Forest plot presenting the standardized mean difference and 95% confidence interval for the impact of nutraceutical supplementation on WOMAC physical function score.

**Figure 11:**
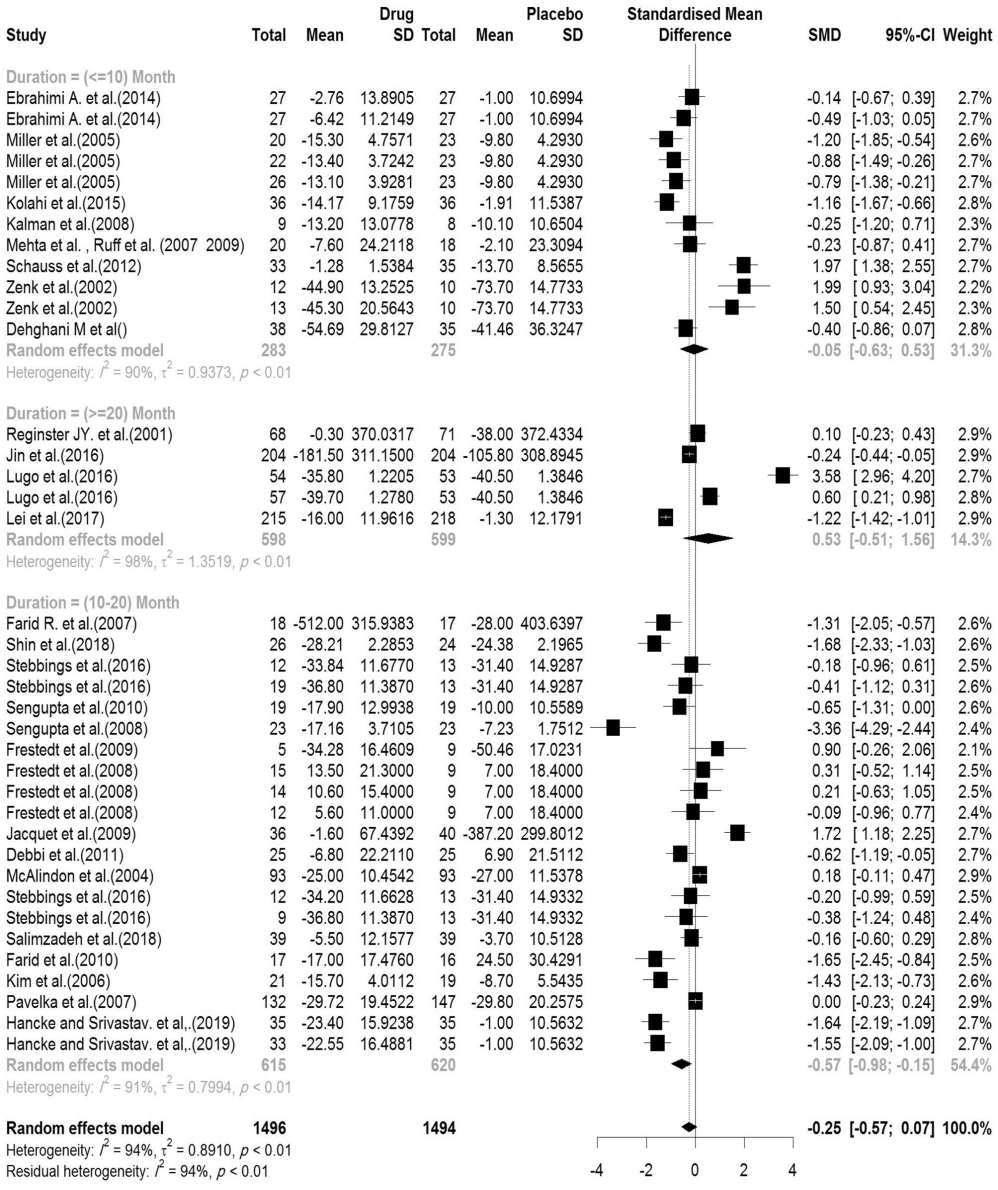
Forest plot presenting the impact of nutraceutical supplementation on WOMAC physical function score (subgroup analysis based on duration of supplementation).

In the included articles, 29 articles assessed the WOMAC Physical Function subscale. There were 1496 subjects in the intervention group and 1494 subjects in the control group. The mean follow-up duration of patients (lowest to maximum) was 7.21 (3–144) weeks. There was a significant heterogeneity between studies (Q-value = 583.74, df = 37, p-value < 0.001, I^2^ = 94.0%) The Pooled Standardized Mean Difference between the intervention and control groups was 0.25 units (SMD = − 0.25, 95% CI − 0.57 to − 0.07, z-value = − 1.55, p-value = 0.12). The forest plot of the combination of results is presented in Fig. 10. The pooled effect size in studies with < 10 months as short term, 10–20 months as medium term and > 20 months as long term supplementation duration were 0.05, 0.57 and 0.53, respectively. The forest plot of the subgroups by the supplementation duration is presented in Fig. 11.

#### Pain (VAS)

**Figure 12:**
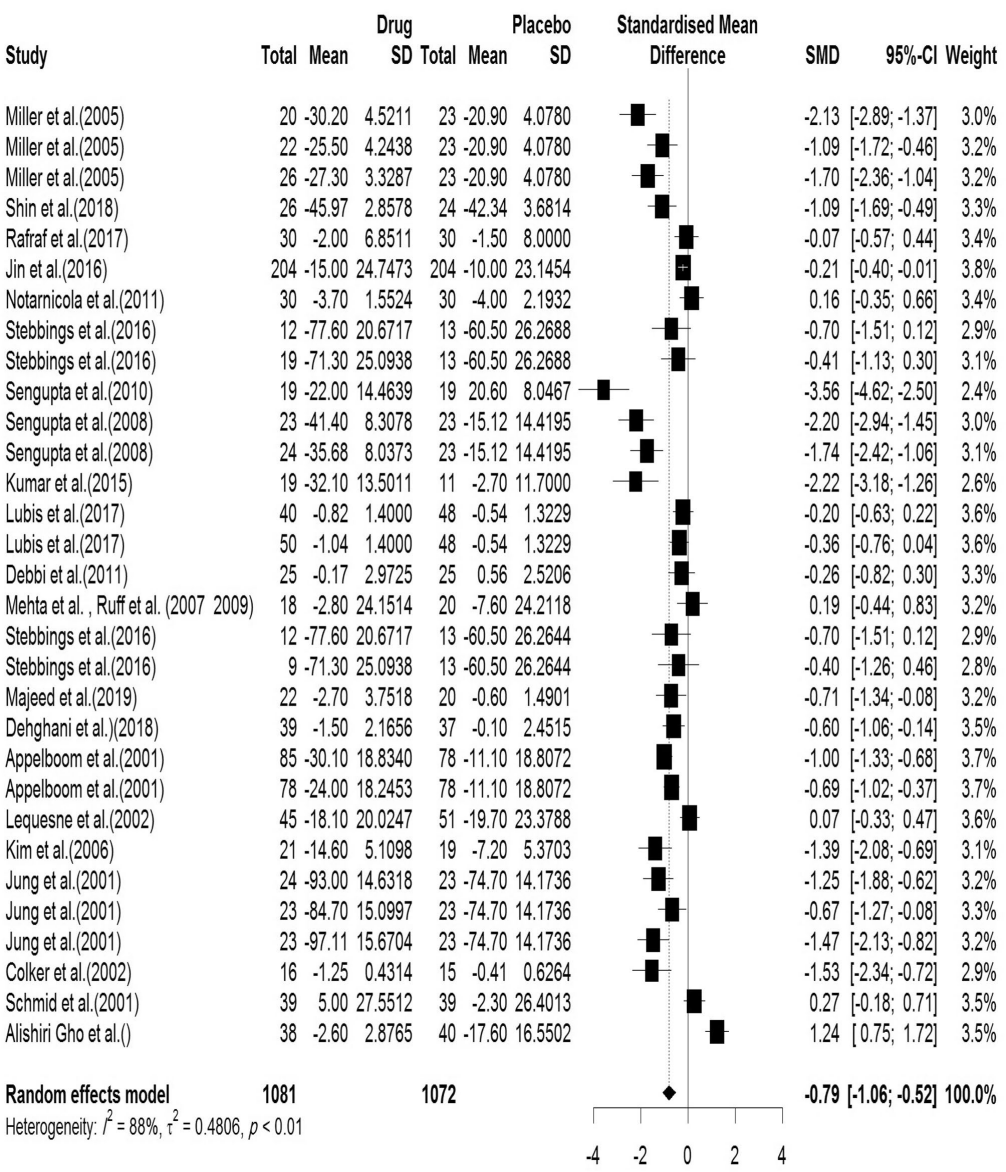
Forest plot presenting the standardized mean difference and 95% confidence interval for the impact of nutraceutical supplementation on VAS.

**Figure 13:**
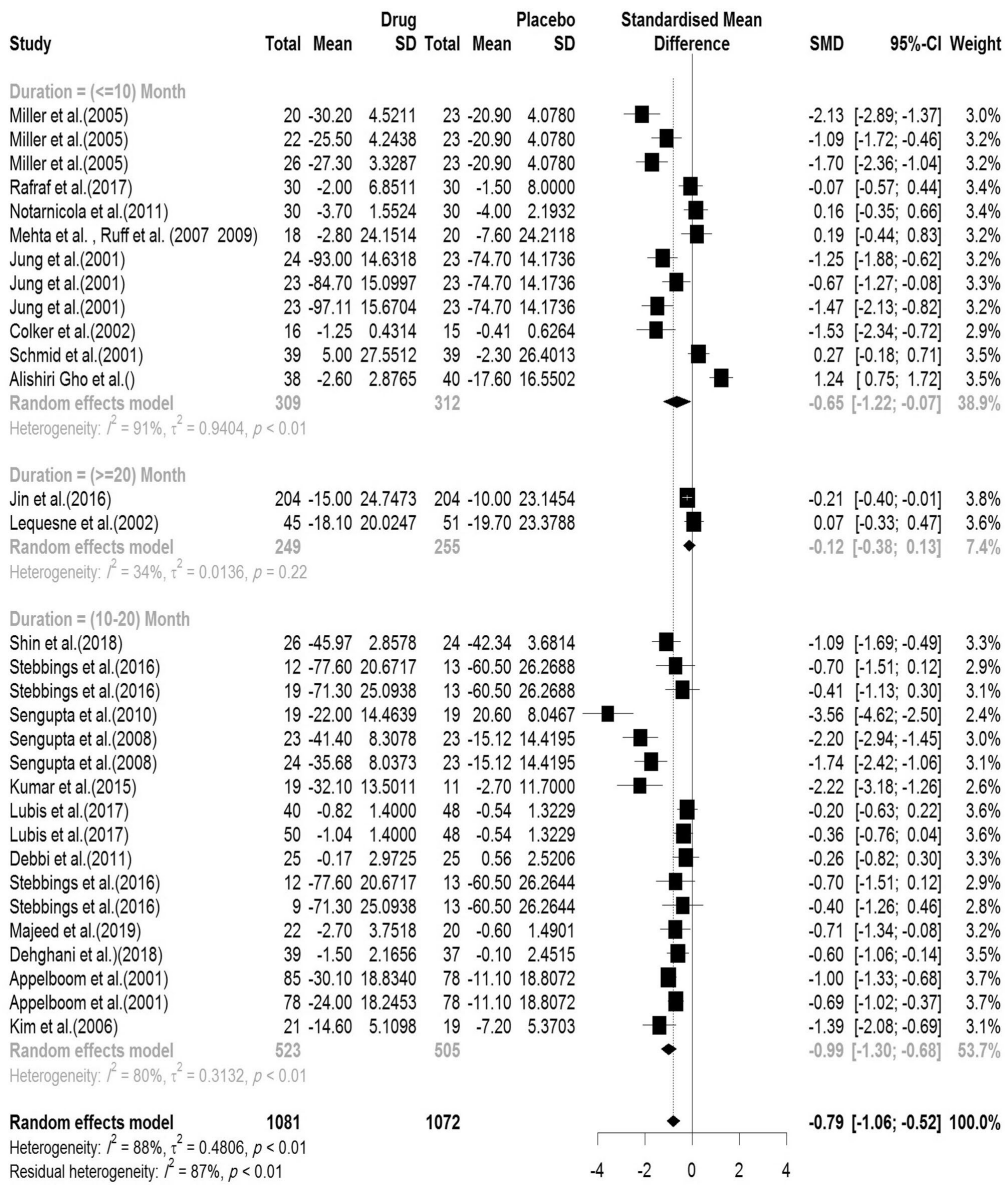
Forest plot presenting the impact of nutraceutical supplementation on VAS (subgroup analysis based on duration of supplementation).

In the included articles, 23 articles assessed the VAS. There were 1081 subjects in the intervention group and 1072 subjects in the control group. The mean follow-up duration of patients (lowest to maximum) was 15.35 (2–96) weeks. There was a significant heterogeneity between studies (Q-value = 246.05, df = 30, p-value < 0.001, I^2^ = 86.5%). The Pooled Standardized Mean Difference between the intervention and control groups was 0.79 units (SMD = − 0.79, 95% CI − 1.06 to − 0.52, z-value = − 5.77, p-value < 0.001). The forest plot of the combination of results is presented in Fig. 12. The pooled effect size in studies with < 10 months as short term, 10–20 months as medium term and > 20 months as long term supplementation duration were 0.65, 0.99 and 0.12, respectively. The forest plot of the subgroups by the supplementation duration is presented in Fig. 13.

### Publication bias for WOMAC index total score

**Figure 14:**
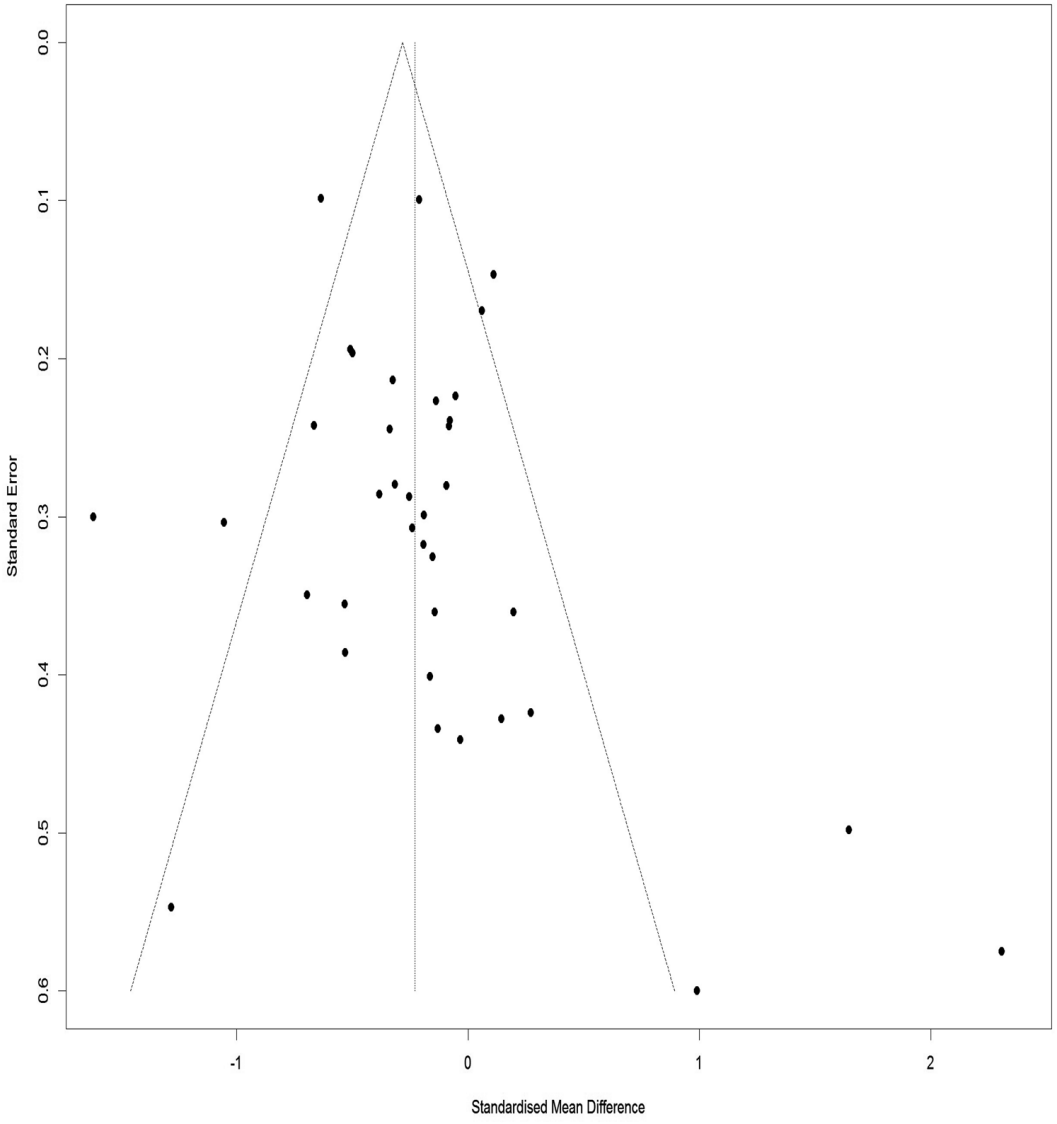
Funnel plot of the publication bias for the WOMAC total score.

Figure 14 illustrates a Funnel Plot to investigate the publication bias for the WOMAC index total score. According to Eggers Regression Test, the publication bias was not significant (t-value = 1.51, df = 36, p-value = 0.13).

### Publication bias for WOMAC index pain subscale

**Figure 15:**
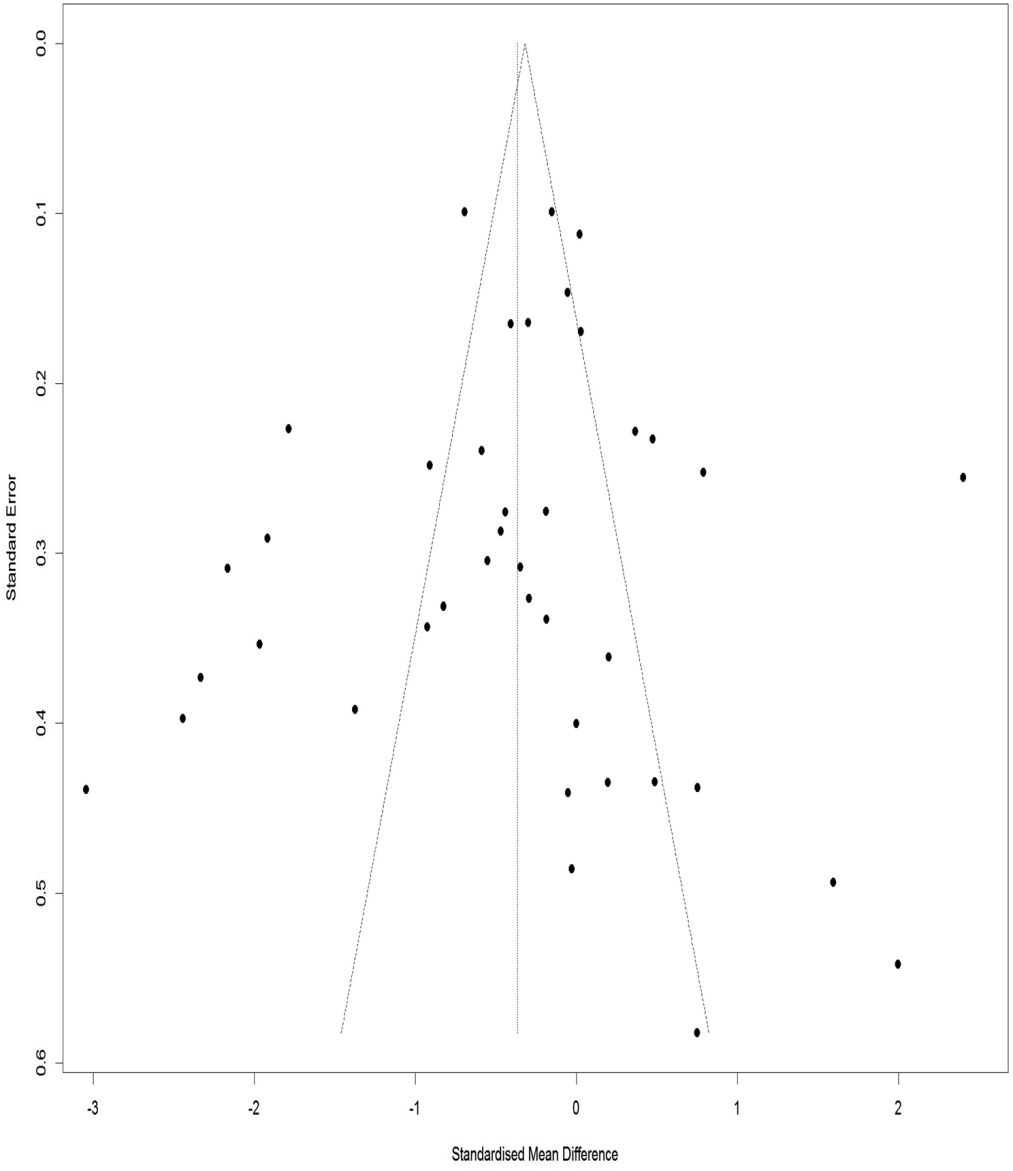
Funnel plot of the publication bias for the WOMAC pain subscale.

Figure 15 illustrates a Funnel Plot to investigate the publication bias for the WOMAC index pain subscale. According to Eggers Regression Test, the publication bias was not significant (t-value = − 0.42, df = 39, p-value = 0.67).

### Publication bias for WOMAC index stiffness subscale

**Figure 16:**
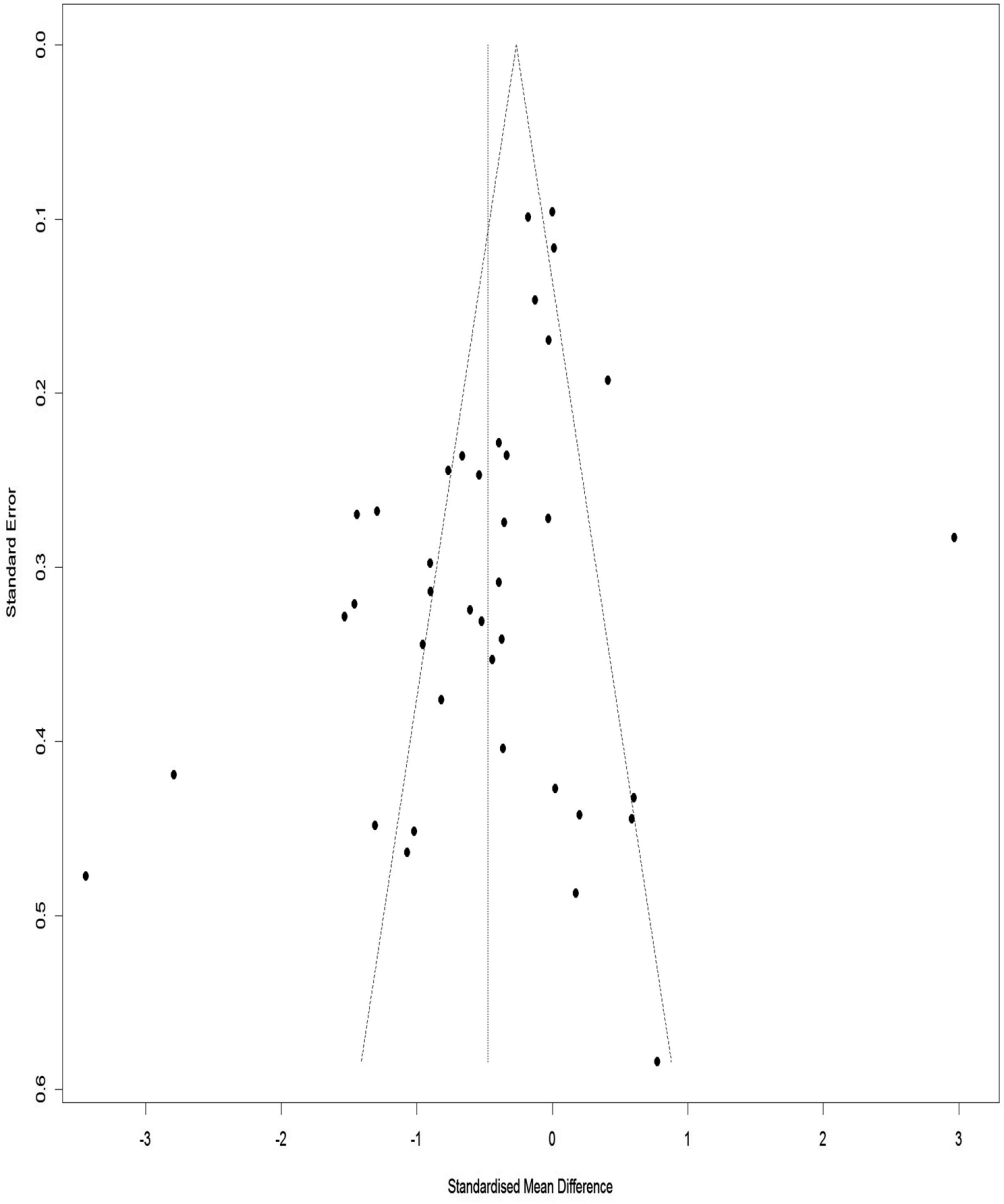
Funnel plot of the publication bias for the WOMAC stiffness subscale.

Figure 16 illustrates a Funnel Plot to investigate the publication bias for the WOMAC index stiffness subscale. According to Eggers Regression Test, the publication bias was significant (t-value = − 2.13, df = 37, p-value = 0.03). Trim and Fill test was performed to modify the publication bias and 11 studies added to adjust for the missed study through this method. The results of the Trim and Fill test demonstrate that the pooled effect size was 0.08 (Adjusted SMD = 0.08, 95% CI − 0.33 to − 0.16).

### Publication bias for WOMAC index physical function subscale

**Figure 17:**
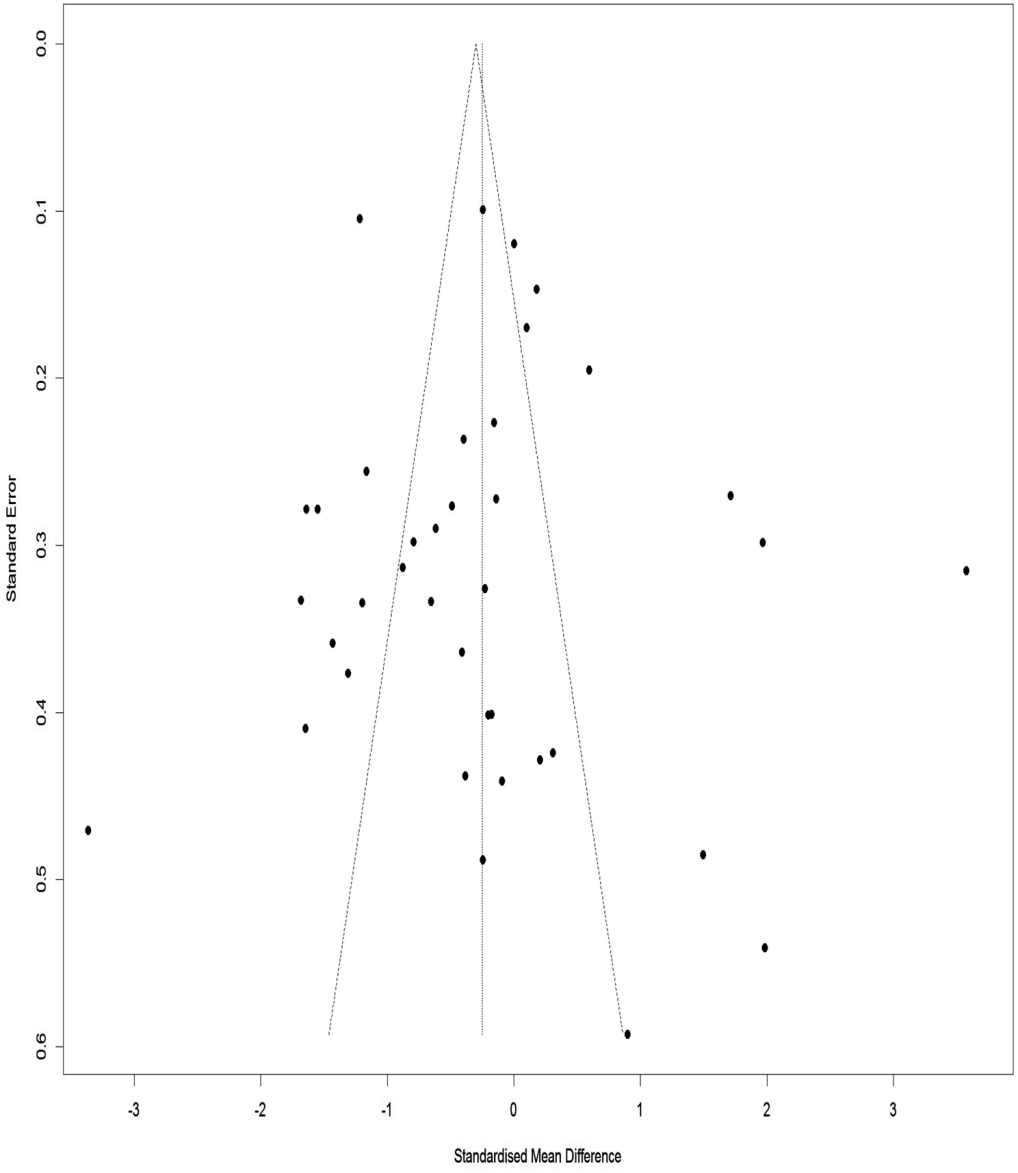
Funnel plot of the publication bias for the WOMAC physical function subscale.

Figure 17 illustrates a Funnel Plot to investigate the publication bias for the WOMAC index physical function subscale. According to Eggers Regression Test, the publication bias was not significant (t-value = − 0.41, df = 39, p-value = 0.68).

### Publication bias for VAS

**Figure 18:**
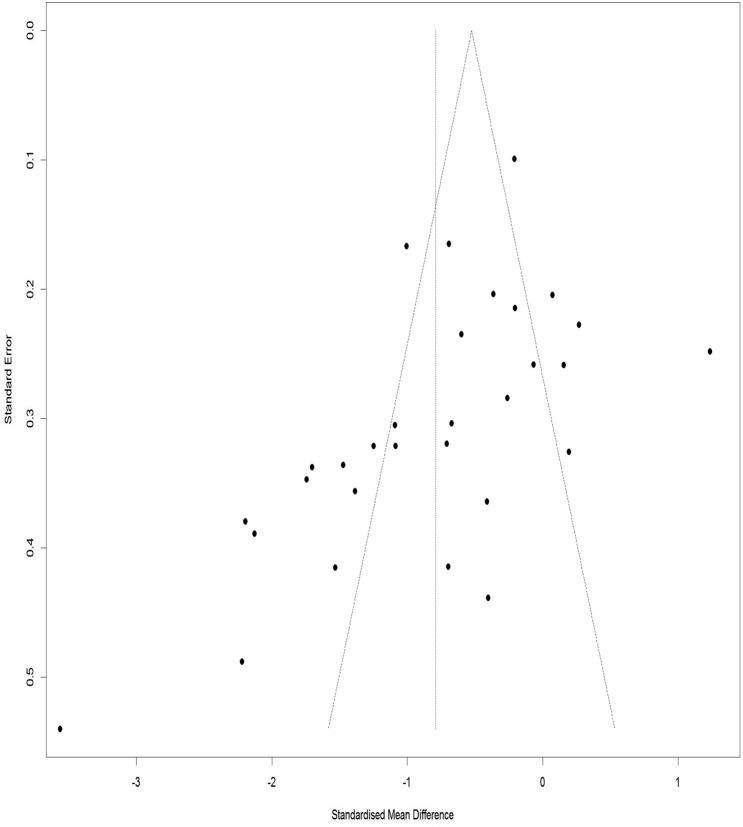
Funnel plot of the publication bias for the VAS.

Figure 18 illustrates a Funnel Plot to investigate the publication bias for the VAS. According to Eggers Regression Test, the publication bias was significant (t-value = − 3.03, df = 29, p-value = 0.004). Trim and Fill test was performed to modify the publication bias and 9 studies added to adjust for the missed study through this method. The results of the Trim and Fill test demonstrate that the pooled effect size was 0.35 (Adjusted SMD = − 0.35, 95% CI − 0.64 to − 0.07).

### Adverse events

The adverse events and dropout rates are summarized in Table 3. The dropout rate ranged from 0 to 41%.

**Table 3:** Adverse events and dropout rate reported by 41 studies.

Author (year)	Dropout rate	Adverse events
Reginster 2001^17^	34% (n = 73)	83 and 101 individuals reported adverse events in GS and placebo group, respectively. No difference was found between treatment and placebo group
Appelboom 2001^18^	13% (n = 35)	28, 24 and 23 individuals reported adverse events in ASU low dose, ASU high dose and placebo group, respectively. No difference was found between treatment and placebo group
Jung 2001^19^	3% (n = 3)	5, 6, 3 and 5 individuals reported adverse events in SKI 306X low dose, SKI 306X medium dose, SKI 306X high dose and placebo group, respectively. No difference was found between treatment and placebo group
Schmid 2001^20^	0	16 and 16 individuals reported adverse events in Willow bark extract and placebo group, respectively. No difference was found between treatment and placebo group
Colker 2002^21^	22% (n = 9)	Adverse events have been supervised. No safety problems were recognized
Zenk 2002^22^	17% (n = 7)	14, 14 and 14 individuals reported adverse events in MPC, GS and placebo group, respectively. No long-term adverse events of any treatment were reported. No difference was found between treatment and placebo group
Lequense 2002^23^	41% (n = 67)	39 and 39 individuals reported adverse events in ASU and placebo group, respectively. No difference was found between treatment and placebo group
McAlindon 2004^24^	9% (n = 19)	18 and 14 individuals reported adverse events in GS and placebo group, respectively. No difference was found between treatment and placebo group
Miller 2005^25^	15% (n = 16)	Adverse events have been supervised. No serious safety problems were recognized
Kim 2006^26^	20% (n = 10)	21 and 19 individuals reported adverse events in MSM and placebo group, respectively. No difference was found between treatment and placebo group
Pavelka 2007^27^	9% (n = 16)	36 and 24 individuals reported adverse events in Diacerein and placebo group, respectively. No statistically significant difference was found between treatment and placebo group
Farid 2007^28^	5% (n = 2)	Adverse events have been supervised. No safety problems were recognized
Mehta 2007^29^	17% (n = 16)	4 and 3 individuals reported adverse events in GS and Reparagen group, respectively. No statistically significant difference was found between ASU groups and the placebo
Alishiri GH.H. 2007^30^	4% (n = 5)	Not report
Sengupta 2008^8^	7% (n = 5)	24, 23 and 23 individuals reported adverse events in 5-Loxin 100, 5-Loxin 250 mg/day and placebo group, respectively. No difference was found between treatment and placebo group
Kalman 2008 ^31^	20% (n = 4)	1 and 2 individuals reported adverse events in Chicken comb extract and placebo group, respectively. No statistically significant difference was found between treatment and placebo group
Frestedt 2008^32^	28% (n = 20)	12, 12, 13 and 140 individuals reported adverse events in Aquamin, GS, GS + Aquamin and placebo group, respectively. No statistically significant difference was found between treatment groups and placebo group
Jacquet 2009^33^	6% (n = 5)	14 and 13 individuals reported adverse events in Phytalgic and placebo group, respectively. No statistically significant difference was found between treatment and placebo group. No statistically significant difference was found between treatment groups and placebo group
Frestedt 2009^34^	36% (n = 8)	8 and 14 individuals reported adverse events in Aquamin and placebo group, respectively
Ruff 2009^35^	37% (n = 22)	Adverse events have been supervised. No safety problems were recognized
Farid 2010^36^	17% (n = 7)	Adverse events have been supervised. No safety problems were recognized
Sengupta 2010^37^	5% (n = 3)	0, 1 and 1 individuals reported adverse events in 5 -Loxin, Aflapin and placebo group, respectively. No statistically significant difference was found between treatment groups and placebo group
Debbi 2011^38^	0	Adverse events have been supervised. No safety problems were recognized
Notarnicola 2011^39^	0	Adverse events have been supervised. No safety problems were recognized
Schauss 2012^40^	15% (n = 12)	3 and 6 individuals reported adverse events in BioCell Collagen and placebo group, respectively. There was no significant difference between the two groups in the total number of adverse events
McAlindon 2013^41^	15% (n = 22)	31 and 23 individuals reported adverse events in Cholecalciferol and placebo group, respectively. There was no significant difference between the two groups in the total number of adverse events
Ebrahimi 2014^42^	13% (n = 12)	Adverse events have been supervised. No safety problems were recognized
Kolahi 2015^43^	4% (n = 3)	Adverse events have been supervised. No safety problems were recognized
Kumar 2015^44^	7% (n = 2)	1 and 0 individuals reported adverse events in PCP and placebo group, respectively. There was no significant difference between the two groups in the total number of adverse events
Dehghan 2015^45^	8% (n = 7)	Not reported
Jin 2016^46^	0	56 and 37 individuals reported adverse events in Vitamin D3 and placebo group, respectively
Stebbings 2016^47^	19% (n = 8)	6, 9 and 7 individuals reported adverse events in ART low dose, ART high dose and placebo group, respectively
Lugo 2016^48^	12% (n = 22)	8, 28 and 9 individuals reported adverse events in UC-II, GC and placebo group, respectively
Lubis 2017^49^	0	Not reported
Rafarf 2017^50^	9% (n = 6)	Not reported
Lei 2017^51^	6% (n = 28)	Adverse events have been supervised. No safety problems were recognized
Shin 2018^52^	17% (n = 10)	Not reported
Dehghani 2018^53^	5% (n = 4)	Not reported
Salimzadeh 2018^54^	5% (n = 4)	Not reported
Hancke 2019^55^	5% (n = 5)	8, 1 and 2 individuals reported adverse events in ParActin low dose, ParActin high dose and placebo group, respectively. There was no significant difference between the ParActin groups and the placebo in the total number of adverse events
Majeed 2019^56^	12% (n = 6)	Adverse events have been supervised. No safety problems were recognized
Rondanelli 2019^57^	0	Adverse events have been supervised. No safety problems were recognized

*ART* Artemisia annua extract, *ASU* Avocado soybean unsaponifiable, *DBE* Deer bone extract, *GC* Glucosamine hydrochloride + chondroitin sulfate, *GS* Glucosamine sulphate, *MSM* Methylsulfonylmethane, *PCP* Collagen peptides isolated from pork skin, *UC-II* Undenatured collagen type II.

## Discussion

This meta-analysis demonstrated that nutraceutical supplementation may lead to an improvement in total and also pain and stiffness subscales of WOMAC and VAS but did not affect WOMAC physical function subscale. The existing modalities for managing OA are basically symptomatic and have not been confirmed to slow, arrest or inverse the joint subversion and cartilage degradation progression^8^. For this reason, over the past few years, attention has been focused on the impact of nutritional supplements in managing and preventing OA, considering its risk–benefit ratio and low cost and great acceptance by patients. Nutraceuticals provide a great variety of products with a broad range of properties such as anti-inflammatory and antioxidant^13,58,59^. Nevertheless, their efficacy in OA is uncertain, yet.

### Short term nutraceutical supplementation in OA patients

In studies with short term duration of supplementation, significant effects of nutraceutical supplement only were seen on VAS and WOMAC stiffness scores. Among these, three supplements [Low dose Sierrasil (2 g/day) in addition to cat's claw extract in patients with mild to moderate knee OA according to Kellgren and Lawrence scoring system for classification of knee OA^60^ and fortified milk-based bioactive micronutrient beverage and SKI 306X in knee OA patients with unspecified disease severity] had significant effects on VAS pain intensity. Low dose Sierrasil in addition to cat's claw extract and l-carnitine had a considerable effect also on WOMAC all subscales in patients with mild to moderate knee OA. Additionally, milk protein concentrate (MPC) showed significant effects on WOMAC stiffness score in knee OA patients with unspecified disease severity and Chicken comb extract with a high content of hyaluronic acid had a considerable effect on WOMAC total score, in patients with mild to severe knee OA according to Kellgren and Lawrence scoring system for classification of knee OA^60^.

Sierrasil is an indigenous mineral product isolated from the Sierra Mountains in the USA with a cultural history of usage in the treatment of joint pain and established cartilage degradation reducing properties^61^. SKI306X is a herbal mixture (Clematis mandshurica, Trichosanthes kirilowii and Prunella vulgaris) applied for the management of inflammatory diseases and is clinically accepted for the treatment of OA in Far East Asia^62^. In the systematic review of RCTs by Ameye and Chee^2^ moderate evidence was established for SKI306X in improving the symptoms in OA patients. Hyaluronic acid or hyaluronan (sodium hyaluronate) is accountable for the viscoelasticity and lubricating impacts of synovial fluid of the joint and has been shown to have the biophysical and biochemical roles in synovial tissues^63^. However, in a recent systematic review and meta-analysis by Liu et al.^64^, collagen hydrolysate, extract of the skin of the passion fruit (PFP), Curcuma longa extract, Boswellia serrata extract, pycnogenol and L-carnitine exhibited clinically important effects for pain alleviation in short term and only two supplements (green-lipped mussel extract and undenatured type II collagen (UC-II) showed clinically important effects on pain reduction at medium term. However, we founded that long term UC- II supplementation had considerable effects on WOMAC total and also WOMAC pain and physical function scale scores in patients with mild Knee OA. UC-II is a natural component which comprises a glycosylated, undenatured type-II collagen. Studies have revealed that UC-II restrain joint health in both OA and rheumatoid arthritis (RA) diseases^48^.

### Medium term nutraceutical supplementation in OA patients

In the subgroup analysis, the greatest efficacy of nutraceutical supplements on WOMAC index total score and its subscales and also VAS was related to medium term supplementation (10 to 20 months). Most of these studies involved patients with mild to moderate knee OA according to Kellgren and Lawrence scoring system for classification of knee OA^60^ or American College of Rheumatology Classification Criteria for Knee Osteoarthritis^65^ which supplements were administered as an adjunctive to symptomatic treatments (NSAIDs and/or analgesics) except nine of them (three^33,47,54^ involved patients with knee and/or hip OA, four^24,32,44,52^ involved patients with severe knee OA and two^53,55^ involved patients for which supplements were administered as a monotherapy and no concomitant treatment were allowed).

Among studies with medium term of supplementation, WOMAC total score was considerably improved through supplementation with CS in patients with mild to moderate knee OA, Deer bone extract (DBE) in patients with moderate to severe knee OA and PFP and collagen peptides isolated from pork skin (PCP) in patients with mild to severe knee OA.

OA is described by damage of type II collagen and GAGs, which are present in the joint. The lessening of GAGs is an essential factor leading to enhanced cartilage deprivation in the OA. CS, a central structural part of cartilage, is a sulfated GAG. Investigations in animal models have suggested that dietary supplements of CS prevent articular cartilage depreciation^66^. This protecting consequence is related to the anti-inflammatory activities of CS by increasing the synthesis of hyaluronic acid and proteoglycans, and decreasing the production of proteolytic enzymes and nitric oxide^57^. Deer horn extract has been considered as a noteworthy health restorative in traditional medicine amongst several Asian countries^67^. Oily DBE and CPC were recently revealed to have anti-inflammatory properties and reduce the morphological deviations related with osteoarthritic cartilage damage in animal models of OA^68,69^.

The WOMAC all subscale scores were improved through medium term supplementation with A. paniculata purified extract (ParActin) (in patients with mild knee OA), DBE (in patients with moderate to severe knee OA) and MSM (in knee OA patients with unknown severity). PFP improved only WOMAC pain and physical function subscales in patients with mild to severe knee OA, Boswellia serrata extract improved only WOMAC pain and stiffness subscales score and VAS in patients with mild to moderate knee OA and Artemisia annua extract (ART) improved considerably only WOMAC stiffness subscale in knee OA with unknown severity.

### Long term nutraceutical supplementation in OA patients

Regarding long term supplementation, skimmed milk containing probiotic Lactobacillus casei Shirota (LcS) had considerably effects on WOMAC total and also WOMAC stiffness scale score and UC- II had considerably effects on WOMAC total and also WOMAC pain and physical function scale scores in patients with mild Knee OA according to Kellgren and Lawrence scoring system for classification of knee OA^60^. Boswellia serrata extract improved WOMAC stiffness scale score in knee OA patients with unspecified disease severity. No supplements were recognized with significant effects on VAS reduction in the long term. However Liu et al.^64^, identified that no supplement had important effects on pain alleviation and physical function improvement in long term in patients with hand, hip or knee OA. These different conclusions are somehow because of different eligibility criteria for included studies and also different scales used for measuring pain and physical function.

There is a growing field of interest and research indicating the protective benefits of dietary polyphenols in decreasing risk for chronic diseases^59^ through accepting electrons from free radicals, distracting chain oxidation reactions, and improving cellular antioxidative capability^16^. The results of several studies suggested that supplementation with polyphenols and botanical extracts (e.g., Boswellia serrata extract, PFP, ParActin, ART and cat's claw extract) decrease the serum levels of TNF-α and MMP-3 in synovial fluid in patients with knee OA compared with the control groups^53,70,71^. Cellular and animal models have suggested also the benefits of such compounds and food ingredients (e.g., probiotics) in inhibiting inflammatory paths and reducing the production of iNOS, COX-2 and MMP enzymes to decrease the catabolic destruction of the cartilage^16,72–76^.

A very important point in our findings which must be considered is that GS and vitamin D with the greatest interest in administration and consumption among OA patients, do not exhibit a clinically significant effect on knee or hip OA. GS is a water-soluble amino monosaccharide, considered as a desired substrate for the biosynthesis of GG chains and is in great amounts in cartilage matrix and synovial fluid. Glucosamine was thought to afford building substrates for the cartilage extracellular matrix biosynthesis. Later studies have established additional clarifications for its anti-inflammatory and anti-catabolic properties. A Cochrane review of RCTs of all GS formulations in OA patients, restricted to studies with satisfactory concealment, failed to display any advantage of GS for pain^77^. Hereafter, GS was firstly suggested by European League Against Rheumatism (EULAR) and Osteoarthritis Research Society International (OARSI) for pain management and structure enhancement in OA patients, but not in the most recent National Institute for Health and Care Excellence (NICE) guidelines.

It has been theorized that vitamin D supplementation in patients with knee OA might be a practicable and cost-effective approach for managing clinical symptoms and making a structural advance. However most clinical trials showed that vitamin D supplementation does not improve cartilage volume or knee pain^41,46,78^. In line with our findings, the results of a systematic review of RCTs covering 1189 patients by Hussein^79^ did not recommend vitamin D supplementation in patients with knee OA.

Our study opens new horizons for the managing of degenerative joint diseases. We collected clinical trials of nutraceuticals and dietary supplements and the findings were really hopeful and encouraging. However, there is a need for more well-designed randomized clinical trials which can confirm the safety and efficacy of such products. This could help clinicians in endorsing them for OA patients.

The present study has some limitations that need to be considered in explicating the results of this systematic review and meta-analysis. Firstly, in spite of an increasing body of nutraceutical researches in subjects with OA, the number of studies included in this specific review after a systematic review of the existing scientific literature was lower than what would have been predicted. We believe that our inclusion criteria had a significant role, because we considered variables (i.e. VAS and WOMAC) that are not measured in many studies. Secondly, there is probable publication bias. Some unpublished abstracts and articles were not included because of unavailability. Thirdly, the language may lead to bias as we selected only the English and Persian language due to limited resources. These may considerably reduce our sample size and accordingly our ability to delineate statistically significant findings. Fourthly, the heterogeneity between the results is an issue need to be considered. Although we did a subgroup analysis, we were not successful to completely minimize these heterogeneities. Finally, there may be some possible aspects not considered in the present systematic review and meta-analysis, such as the severity of OA, region, and race.

In spite of the stated limitations, this systematic review and meta-analysis provides the first systematic work to consider clinical trials on nutraceutical supplementation in relation to pain and physical disability in patients with knee/hip OA. In addition, subgroup analysis was implemented according to the nutraceutical type and we applied more suitable consequence indicators to direct this meta-analysis.

In conclusion, nutraceutical supplementation mostly along with symptomatic treatments (NSAIDs/ COX-2 inhibitors and analgesics) may effectively improve pain and physical function in patients with knee/hip OA. In the subgroup analysis, the greatest efficacy of nutraceutical supplements was related to 10–20 month (medium term) supplementation especially in patients with mild to severe knee OA. Despite recognized supplements with no established significant efficacy in our study (such as glucosamine and vitamin D), some not well-known supplements (Boswellia serrata extract, DBE, PFP, PCP, ParActin, ART and Pycnogenol) seem to have largest benefits in decreasing pain and improving physical function with negligible adverse events. It is recommended to trying these supplements in a safe doses along with conventional symptomatic treatments and physical therapy for at least 10 weeks especially for those with mild to moderate knee OA except low dose Sierrasil in addition to cat's claw extract, fortified bioactive micronutrient beverage, SKI 306X, L-carnitine, MPC and hyaluronic acid which are expected to have beneficial effects in decreasing pain and/or disability in less than 10 weeks of supplementation and also probiotic LcS and UC-II which are not anticipated to have favorable effects in less than 20 weeks of supplementation even in patients with mild knee OA. Other more precise outcome measurement tools, such as inflammatory biomarkers or image study, should probably be introduced into future studies to make them more convincing evidence.
